# Global burden of lip and oral cavity cancer attributable to high alcohol consumption from 1990 to 2021

**DOI:** 10.3389/fnut.2025.1648788

**Published:** 2025-09-29

**Authors:** Shimeng Pang, Wei Duan, Wei Chen, Le Lu

**Affiliations:** Suzhou Stomatological Hospital, Suzhou, China

**Keywords:** lip and oral cavity cancer, high alcohol consumption, global burden of disease, DALYs, SDI, epidemiological trends

## Abstract

**Background:**

Based on data from the Global Burden of Disease (GBD) 2021, this study analyzes the global disease burden of lip and oral cavity cancer attributable to high alcohol consumption (LOC-HAC).

**Methods:**

Disability-adjusted life years (DALYs) were used to assess health loss. The Das Gupta decomposition method was employed to analyze the effects of population growth, aging, and epidemiological changes on DALYs. The Nordpred model was applied to project the disease burden trends from 2022 to 2045.

**Results:**

In 2021, the global number of DALY cases for LOC-HAC was 1,191,084 (95% UI: 906,229.2–1,474,007), representing a 90% increase from 628,484.1 (95% UI: 493,522.1–754,895.3) in 1990. The fastest growth occurred in low-middle SDI regions, reaching 279%. A similar increasing trend was observed in deaths, with Southeast Asia showing the most significant rise, where DALYs and deaths increased by 326 and 357%, respectively. The global age-standardized DALY rate (ASDR) of LOC-HAC in 2021 was 13.66 (95% UI: 10.4–16.9) per 100,000 population, with Central Europe having the highest ASDR and Southeast Asia experiencing the fastest growth. DALYs and deaths were significantly higher in males than in females and increased with age, peaking at 55–59 years in males and 60–64 years in females. SDI levels were positively correlated with ASDR and age-standardized mortality rates (ASMR). However, when the SDI exceeded 0.75, ASDR and ASMR showed a declining trend. Over the past 32 years, the global increase in DALYs and deaths has been primarily driven by population aging and growth. Projections indicate that from 2022 to 2045, DALYs and deaths among males will rise significantly, whereas the increase among females will be more gradual.

**Conclusion:**

The global burden of LOC-HAC has risen significantly over the past decades, especially in low-middle SDI regions and Southeast Asia. In the future, efforts should focus on these regions by promoting healthier lifestyles, strengthening early screening, and optimizing healthcare resource allocation to reduce disease burden.

## Introduction

Lip and oral cavity cancer (LOC) is one of the more common malignant tumors worldwide (1). In recent years, its incidence has shown a significant upward trend, imposing a substantial burden on global healthcare systems (2). Major risk factors for LOC include tobacco smoking, alcohol consumption, betel nut chewing, human papillomavirus (HPV) infection, and ultraviolet (UV) exposure (2). HPV infection is associated with an increased risk of oral cancer, while UV exposure primarily elevates the risk of lip cancer (3). Therefore, there is an urgent need for comprehensive global research to better understand the epidemiological patterns of LOC and to develop targeted prevention strategies aimed at reducing its incidence and mortality.

In 2020, approximately 741,300 new cancer cases globally were attributed to alcohol consumption, accounting for 4% of all newly diagnosed cancer cases that year (4). A strong association exists between heavy alcohol consumption and the risk of LOC. Studies have shown a positive correlation between alcohol consumption and the risk of oral cancer. For instance, moderate drinkers (more than 12.5 g but less than or equal to 50 g of alcohol per day) have a 1.8-fold higher risk of developing oral cancer compared to non-drinkers, while heavy drinkers (more than 50 g per day) face a fivefold increased risk (5).

Studies have shown that alcohol consumption can lead to LOC through multiple mechanisms (6–9). First, ethanol metabolism in the body produces acetaldehyde, a known carcinogen that can bind to DNA to form adducts, causing DNA damage and mutations, thereby increasing the risk of malignant transformation (10). In addition, ethanol metabolism generates large amounts of reactive oxygen species (ROS), which induce oxidative stress, lipid peroxidation, and DNA damage, while also disrupting cell signaling pathways to promote cell proliferation and metastasis (11). Ethanol can also interfere with DNA methylation and epigenetic modifications, leading to abnormal gene expression and further promoting carcinogenesis (12). Chronic heavy drinking impairs immune system function, allowing malignant cells to escape immune surveillance (13). Moreover, alcohol acts synergistically with other carcinogenic factors, such as tobacco, significantly increasing the risk of LOC (14).

This study, based on data from the Global Burden of Disease Study 2021 (GBD 2021), provides an in-depth analysis of the epidemiological characteristics of lip and oral cavity cancer attributable to high alcohol consumption (LOC-HAC). The research focuses on temporal trends in the age-standardized mortality rates (ASMR) and the age-standardized DALY rates (ASDR) associated with LOC-HAC, revealing its dynamic changes over time. Further analysis shows that gender, age, and Socio-demographic Index (SDI) significantly influence the burden of LOC across different regions and populations. In addition, the study evaluates the contributions of aging, population growth, and epidemiological changes to the LOC-HAC. It also assesses the extent of inequality among countries and projects future trends through 2045. The findings provide critical scientific evidence for the development of public health policies and initiatives aimed at reducing the burden of LOC-HAC, supporting policymakers in implementing targeted strategies to improve global public health outcomes.

## Methods

### Data sources and disease definition

This study was based on data from the GBD 2021 to analyze the burden of LOC-HAC. LOC refers to malignant tumors of the lip and oral cavity, with ICD-10 codes C00–C08 (3, 15). Disability-adjusted life years (DALYs) were used as the primary measure of health loss, consisting of years of life lost (YLLs) and years lived with disability (YLDs), thereby quantifying the overall disease burden (16–19). Uncertainty intervals (UIs) were used to reflect the reliability of the estimates, generally reported as 95% UIs (16, 20). This means there is a 95% probability that the true value falls within the given range, calculated from the 2.5th and 97.5th percentiles of 1,000 posterior draws. This approach accounts for differences in estimation methods across countries and uncertainties from multiple imputations of missing values. Uncertainty is propagated at each step to ensure that the final UIs fully capture estimation variability. Alcohol consumption was identified as a modifiable behavioral risk factor contributing to the burden of LOC. In GBD 2021, alcohol intake was assessed based on population-based survey data of self-reported consumption. Harmful drinking was defined as alcohol consumption exceeding the theoretical minimum risk exposure level—the level associated with the lowest risk of overall health loss (21).

### Decomposition analysis and cross-country inequality analysis

To explore the drivers of changes in disease burden, the Das Gupta decomposition method was applied (22). This statistical technique, widely used in GBD studies, decomposes observed changes in disease burden indicators (e.g., mortality, DALYs) into three independent components: aging, population growth, and epidemiological changes (23). Additionally, cross-country inequality analysis was conducted using the slope index of inequality (SII) and concentration index (CI) to assess socioeconomic disparities in disease burden across countries worldwide (24).

### Forecasting analysis

The Nordpred model is a generalized linear model based on the age–period–cohort (APC) framework, widely used to predict epidemiological indicators such as DALY rates and mortality (25, 26). Built upon generalized linear modeling, it incorporates age, period, and cohort effects, and applies linear extrapolation to estimate future disease burden. The mathematical form of the model can be expressed as: log(μ_ijk_) = *α* + β_i_*Age_i_ + γ_j_*Period_j_ + δ_k_*Cohort_k_, where μ_ijk_ represents the ASDR/ASMR for a specific age group, period, and birth cohort; *α* is the intercept; and β_i_, γ_j_, and δ_k_ denote the regression coefficients for age, period, and cohort effects, respectively (26–28).

In this study, we used the Nordpred package in R software (version 4.2.1) to perform APC modeling and predict the future burden of LOC-HAC. Age groups were defined in 5-year intervals, and the prediction horizon was set to 24 years. To address uncertainties in future trends, we applied the default trend attenuation scheme of the model to improve robustness.

## Results

### Global and regional burden of LOC-HAC

In 2021, the global number of DALYs cases from LOC-HAC was 1,191,084 (95% UI: 906,229.2–1,474,007), representing a 90% increase compared with 628,484.1 (95% UI: 493,522.1–754,895.3) in 1990. Middle SDI regions had the highest number of DALYs cases, 372,549.9 (95% UI: 278,922.6–468,908.9), with a percentage change of 192%. The fastest growth occurred in low-middle SDI regions, with a percentage change of 279%. In 2021, global death cases reached 40,038.13 (95% UI: 30,762.25–49,621.09), a 102% increase compared with 19,863.53 (95% UI: 15,571.83–24,071.57) in 1990. Middle SDI regions recorded the highest number of death cases at 12,017.44 (95% UI: 9012.97–15,122.74), with a percentage change of 215%. The fastest growth in death cases occurred in low-middle SDI regions, at 296%. Over the past 32 years, Southeast Asia had the fastest growth among the 21 GBD regions, with DALYs and death cases increasing by 326 and 357%, respectively (Tables 1, 2).

**Table 1:** Disability-Adjusted Life Years (DALYs) and age-standardized DALY rate (ASDR) of lip and oral cavity cancer attributable to high alcohol consumption in 1990 and 2021, and the PC and EAPC from 1990 to 2021.

Location	1990_DALYs cases (95% UI)	2021_DALYs cases (95% UI)	Percentage change	1990_ASDR_per 100,000(95% UI)	2021_ASDR_per 100,000(95% UI)	EAPC (95% CI)
Andean Latin America	1168.25 (713.59–1582.54)	2849.44 (2020.49–3910.61)	1.44	4.94 (3.02–6.75)	4.56 (3.23–6.28)	0.08 (−0.18–0.33)
Australasia	4366.86 (3167.81–5508.4)	6535.35 (5082.98–8055.99)	0.5	19.31 (14.16–24.28)	13.72 (10.69–16.78)	−0.99 (−1.19--0.79)
Caribbean	3872.46 (2914.13–4854.68)	7530.72 (5625.16–9578.66)	0.94	14.27 (10.65–17.95)	13.99 (10.46–17.75)	0.21 (0.1–0.31)
Central Asia	6837.54 (4998.3–8663.17)	8979.08 (6439.28–11493.38)	0.31	13.07 (9.52–16.63)	9.48 (6.73–12.21)	−1.11 (−1.3--0.92)
Central Europe	50125.19 (39886.79–59707.18)	64672.17 (52041.72–76742.81)	0.29	34.02 (27.21–40.44)	34.5 (27.77–40.93)	−0.11 (−0.24–0.02)
Central Latin America	6342.39 (4922.02–7709.53)	12686.71 (9429.43–16026.72)	1	6.59 (5.04–8.03)	4.86 (3.62–6.14)	−1.31 (−1.48--1.14)
Central Sub-Saharan Africa	1744.9 (659.11–2778.3)	5438.1 (2785.12–8032.37)	2.12	6.44 (2.58–10.14)	7.69 (3.97–11.37)	1.14 (0.48–1.79)
East Asia	76927.84 (56636.47–97120.57)	200464.2 (145112.2–267452.4)	1.61	7.77 (5.7–9.85)	9.07 (6.6–12.1)	0.76 (0.49–1.03)
Eastern Europe	85125.38 (65673.11–101773.5)	95239.42 (71064.14–115200.9)	0.12	30.59 (23.89–36.46)	30.39 (23.04–36.78)	−0.5 (−0.74--0.26)
Eastern Sub-Saharan Africa	10269.95 (5118.22–13417.59)	28801.63 (18267.69–39777.77)	1.8	11.42 (5.93–14.97)	13.47 (8.72–18.56)	0.4 (0.25–0.55)
Global	628484.1 (493522.1–754895.3)	1,191,084 (906229.2–1,474,007)	0.9	14.73 (11.59–17.72)	13.66 (10.4–16.9)	−0.25 (−0.33--0.17)
High-income Asia Pacific	18457.3 (14813.79–21895.05)	29593.93 (22464.6–35783.15)	0.6	8.97 (7.19–10.64)	8.23 (6.42–9.9)	−0.78 (−1.16--0.4)
High-income North America	49404.75 (35197.27–63976.66)	65133.2 (50207.83–79856.59)	0.32	15.56 (11.27–20.01)	11.01 (8.59–13.39)	−0.91 (−1.11--0.71)
High-middle SDI	206857.3 (164591.7–245929.7)	265722.5 (210203.5–323951.6)	0.28	19.84 (15.78–23.61)	13.81 (10.99–16.81)	−1.39 (−1.48--1.3)
High SDI	211459.3 (168610.7–251979.7)	249243.5 (197586.3–296439.2)	0.18	20.48 (16.41–24.33)	13.95 (11.16–16.54)	−1.26 (−1.3--1.23)
Low-middle SDI	61926.3 (31162.55–87152.28)	234938.5 (157314.2–315269.2)	2.79	8.38 (4.14–11.75)	14.31 (9.61–19.18)	2.1 (1.88–2.32)
Low SDI	19585.7 (9718.81–27394.52)	67165.81 (41626.59–91328.5)	2.43	7.14 (3.55–9.96)	10.73 (6.64–14.73)	1.43 (1.18–1.69)
Middle SDI	127548.9 (94638.94–157670.2)	372549.9 (278922.6–468908.9)	1.92	10.45 (7.73–12.94)	13.04 (9.77–16.4)	0.83 (0.69–0.96)
North Africa and Middle East	1652.64 (1101.73–2316.17)	3381.04 (2258.39–4593.56)	1.05	0.79 (0.5–1.11)	0.62 (0.39–0.85)	−1.04 (−1.16--0.91)
Oceania	128.39 (65.38–194.81)	359.13 (207.03–526.91)	1.8	3.15 (1.55–4.78)	3.49 (1.94–5.16)	0.75 (0.45–1.05)
South Asia	97834.64 (43488.68–138268.7)	375737.6 (250624.1–515357.1)	2.84	13.57 (5.99–19.18)	22.38 (14.81–30.73)	1.92 (1.69–2.15)
Southeast Asia	18389.46 (12758.74–23392.93)	78422.75 (58547.35–99565.23)	3.26	6.04 (4.2–7.65)	10.68 (7.95–13.55)	2.03 (1.91–2.14)
Southern Latin America	9012.61 (7313.42–10898.99)	8612.95 (6555.64–10684.11)	−0.04	19.23 (15.6–23.2)	10.39 (7.95–12.91)	−1.62 (−1.82--1.41)
Southern Sub-Saharan Africa	7970.34 (4994.93–10556.31)	14668.43 (10360.3–18388.69)	0.84	25.67 (15.89–34.17)	21.97 (15.51–27.37)	−0.9 (−1.11--0.69)
Tropical Latin America	18658.11 (14778.78–22949.39)	43435.09 (33720.95–53663.59)	1.33	17.62 (13.94–21.71)	16.32 (12.65–20.16)	−0.39 (−0.68--0.09)
Western Europe	157171.9 (126863.1–184811.7)	128,077 (103333.6–151797.2)	−0.19	31.24 (25.42–36.69)	16.82 (13.66–20.11)	−2.08 (−2.14--2.02)
Western Sub-Saharan Africa	3023.17 (1814.01–4012.61)	10466.57 (6999–13749.09)	2.46	2.99 (1.83–3.99)	4.27 (2.89–5.56)	1.06 (0.98–1.14)

**Table 2:** Deaths and age-standardized mortality rate (ASMR) of lip and oral cavity cancer attributable to high alcohol consumption in 1990 and 2021, and the PC and EAPC from 1990 to 2021.

Location	1990_Death cases (95% UI)	2021_Death cases (95% UI)	Percentage change	1990_ASMR_per 100,000(95% UI)	2021_ASMR_per 100,000(95% UI)	EAPC (95% CI)
Andean Latin America	36.12 (22.22–49.6)	94.44 (66.19–132.13)	1.61	0.168 (0.103–0.232)	0.157 (0.109–0.221)	0.16 (−0.12–0.43)
Australasia	152.03 (99.96–197.22)	259.75 (196.39–324.45)	0.71	0.66 (0.44–0.852)	0.494 (0.377–0.611)	−0.8 (−1.02--0.57)
Caribbean	129.22 (94.58–164.33)	259.69 (187.7–331.29)	1.01	0.492 (0.357–0.629)	0.48 (0.348–0.612)	0.22 (0.11–0.33)
Central Asia	201.81 (143.55–258.75)	276.45 (191.62–358.04)	0.37	0.4 (0.281–0.517)	0.309 (0.207–0.406)	−0.83 (−1.04--0.62)
Central Europe	1579.97 (1242.7–1905.82)	2270.33 (1808.4–2690.69)	0.44	1.067 (0.835–1.286)	1.13 (0.905–1.343)	0.09 (−0.01–0.19)
Central Latin America	202.44 (153.73–248.89)	429.04 (316.05–549.94)	1.12	0.236 (0.178–0.293)	0.169 (0.124–0.217)	−1.38 (−1.55--1.21)
Central Sub-Saharan Africa	53.32 (21.05–84.19)	161.32 (83.24–238.11)	2.03	0.227 (0.098–0.353)	0.267 (0.143–0.392)	1.1 (0.41–1.8)
East Asia	2413.52 (1764.92–3077.47)	7033.81 (5061.39–9471.98)	1.91	0.266 (0.193–0.338)	0.319 (0.231–0.43)	0.87 (0.57–1.17)
Eastern Europe	2563.96 (1890.04–3114.93)	2969.02 (2113.25–3654.22)	0.16	0.907 (0.672–1.103)	0.903 (0.655–1.104)	−0.47 (−0.72--0.22)
Eastern Sub-Saharan Africa	311.94 (164.11–409.99)	858.01 (556.72–1178.01)	1.75	0.388 (0.212–0.512)	0.462 (0.307–0.624)	0.45 (0.29–0.62)
Global	19863.53 (15571.83–24071.57)	40038.13 (30762.25–49621.09)	1.02	0.489 (0.381–0.594)	0.462 (0.354–0.572)	−0.17 (−0.26--0.08)
High-income Asia Pacific	622.42 (490.47–744.94)	1381.36 (1006.9–1722.26)	1.22	0.309 (0.243–0.371)	0.302 (0.23–0.367)	−0.6 (−0.98--0.22)
High-income North America	1667.83 (1109.27–2252.22)	2521.09 (1904.02–3127.61)	0.51	0.503 (0.343–0.671)	0.393 (0.299–0.486)	−0.56 (−0.77--0.35)
High-middle SDI	6567.5 (5134.12–7900.38)	9117.8 (7038.96–11166.48)	0.39	0.647 (0.501–0.781)	0.464 (0.359–0.568)	−1.24 (−1.32--1.16)
High SDI	7072.46 (5446.4–8596.82)	9729.96 (7737.17–11631.72)	0.38	0.665 (0.516–0.806)	0.49 (0.39–0.585)	−0.99 (−1.03--0.94)
Low-middle SDI	1796.55 (872.08–2511.16)	7113.04 (4745.81–9548.7)	2.96	0.264 (0.129–0.371)	0.462 (0.306–0.621)	2.15 (1.95–2.36)
Low SDI	582.28 (293.31–815.42)	2008.67 (1235.24–2768.73)	2.45	0.234 (0.119–0.325)	0.362 (0.222–0.502)	1.55 (1.27–1.82)
Middle SDI	3809.36 (2826.42–4728.74)	12017.44 (9012.97–15122.74)	2.15	0.342 (0.255–0.427)	0.433 (0.325–0.546)	0.88 (0.74–1.03)
North Africa and Middle East	46.99 (29.61–65.61)	100.97 (63.2–139.57)	1.15	0.025 (0.016–0.035)	0.021 (0.012–0.029)	−0.92 (−1.06--0.78)
Oceania	3.37 (1.66–5.09)	9.62 (5.35–14.24)	1.85	0.096 (0.047–0.144)	0.107 (0.06–0.16)	0.81 (0.49–1.12)
South Asia	2769.49 (1214.95–3934.21)	11251.97 (7275.53–15519.07)	3.06	0.419 (0.186–0.592)	0.71 (0.452–0.983)	1.96 (1.73–2.2)
Southeast Asia	550.71 (383.07–696.6)	2515.37 (1872.14–3224.78)	3.57	0.203 (0.139–0.258)	0.368 (0.273–0.468)	2.15 (2.02–2.28)
Southern Latin America	305.92 (246.71–373.27)	316.11 (236.08–392.54)	0.03	0.659 (0.53–0.806)	0.369 (0.276–0.456)	−1.45 (−1.65--1.26)
Southern Sub-Saharan Africa	238.97 (147.13–319.19)	447.24 (315.56–559.06)	0.87	0.831 (0.503–1.115)	0.719 (0.507–0.898)	−0.85 (−1.06--0.64)
Tropical Latin America	556.56 (440.34–689.94)	1407.16 (1086.73–1752.43)	1.53	0.564 (0.447–0.705)	0.535 (0.412–0.667)	−0.26 (−0.54–0.02)
Western Europe	5362.56 (4172.52–6374.97)	5157.58 (4061.56–6187.76)	−0.04	1.005 (0.792–1.191)	0.595 (0.474–0.709)	−1.73 (−1.79--1.67)
Western Sub-Saharan Africa	94.38 (58.04–125.7)	317.8 (215.57–414.21)	2.37	0.103 (0.064–0.138)	0.149 (0.102–0.192)	1.13 (1.06–1.2)

**Figure 1:**
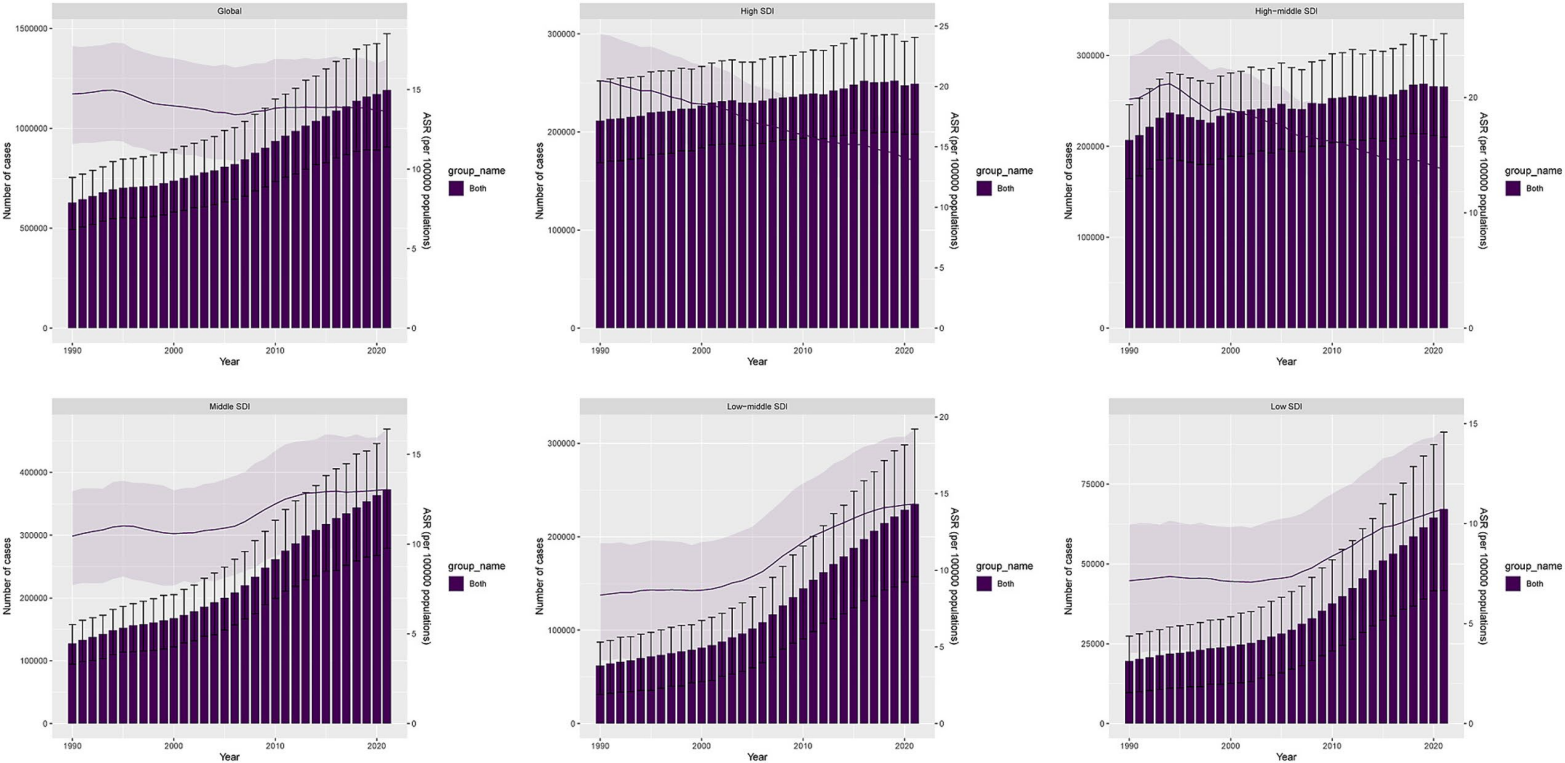
DALYs cases and ASDR of lip and oral cavity cancer attributable to high alcohol consumption from 1990 to 2021.

In 2021, the global ASDR of LOC-HAC was 13.66 (95% UI: 10.4–16.9) per 100,000 population, with an EAPC of −0.25 (95% CI: −0.33 to −0.17). The highest ASDR was in Central Europe at 34.5 (95% UI: 27.77–40.93) per 100,000. The fastest increase in ASDR occurred in Southeast Asia, with an EAPC of 2.03 (95% CI: 1.91–2.14) (Figure 1). The global ASMR of LOC-HAC was 0.462 (95% UI: 0.354–0.572) per 100,000, with an EAPC of −0.17 (95% CI: −0.26 to −0.08). The highest ASMR was in Central Europe at 1.13 (95% UI: 0.905–1.343) per 100,000. The fastest growth in ASMR was also in Southeast Asia, with an EAPC of 2.15 (95% CI: 2.02–2.28) (Supplementary Figure 1).

### National Burden of LOC-HAC

**Figure 2:**
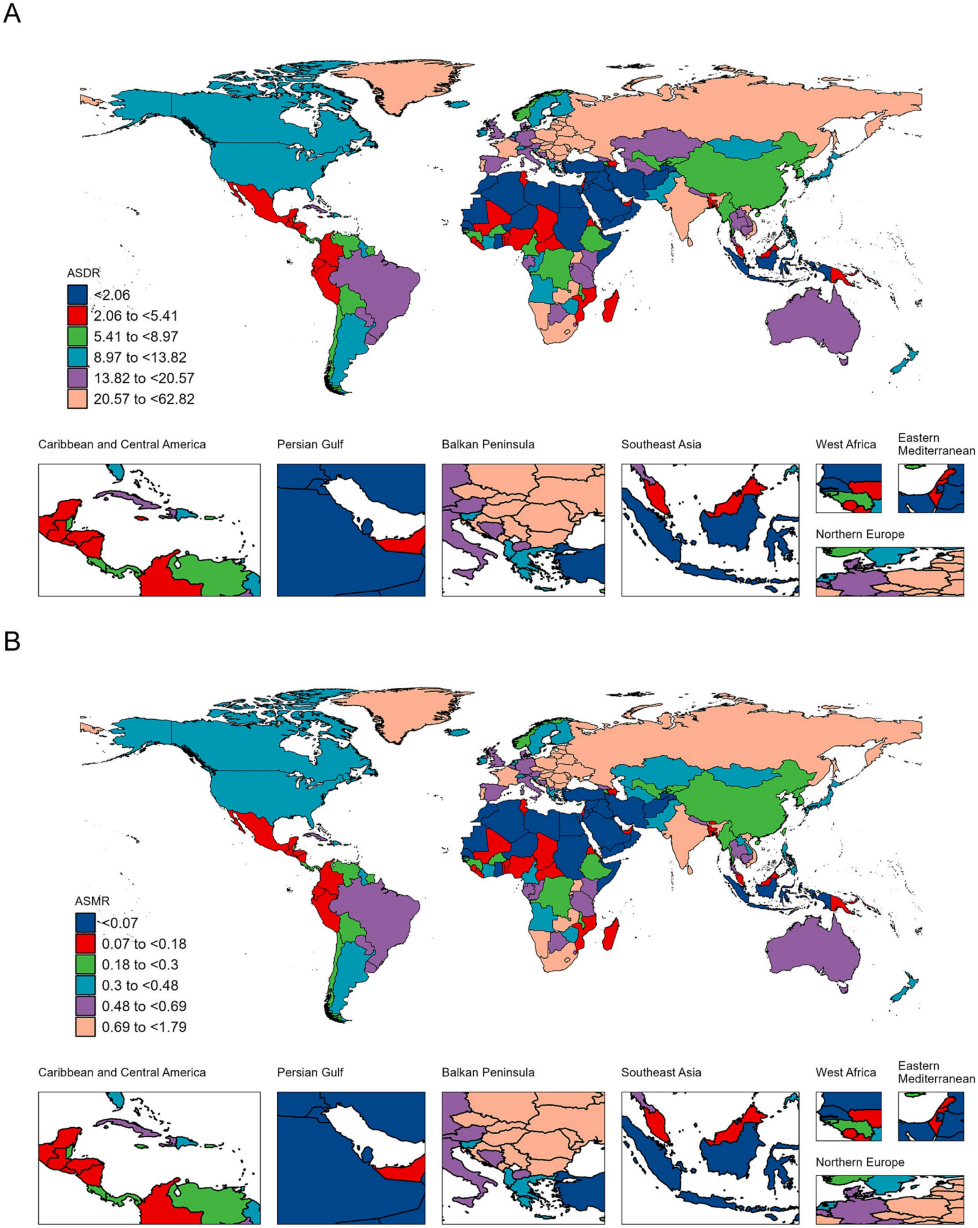
Maps depicting the ASDR **(A)** and ASMR **(B)** of lip and oral cavity cancer attributable to high alcohol consumption across 204 countries and territories in 2021.

From 1990 to 2021, the five countries with the fastest growth in DALYs cases were Iran, Libya, Cape Verde, Viet Nam, and Nepal, with percentage changes of 1,536,100%, 30,485%, 6,955%, 6,924%, and 3,849%, respectively. The five countries with the fastest growth in ASDR were Iran, Libya, Viet Nam, Nepal, and Cape Verde, with EAPCs of 38.43 (95% CI: 31.22–46.04), 11.54 (95% CI: 7.48–15.75), 10.96 (95% CI: 9.47–12.47), 9.44 (95% CI: 8.11–10.78), and 9.05 (95% CI: 6.45–11.71), respectively (Figure 2A and Supplementary Table 1).

The five countries with the fastest growth in death cases were Viet Nam, Cape Verde, Nepal, Myanmar, and Cambodia, with percentage changes of 7,526%, 6,120%, 4,403%, 1,501%, and 1,289%, respectively. The five countries with the fastest growth in ASMR were Iran, Libya, Viet Nam, Nepal, and Cape Verde, with EAPCs of 41.04 (95% CI: 33.18–49.36), 11.94 (95% CI: 7.71–16.34), 11.26 (95% CI: 9.72–12.83), 9.77 (95% CI: 8.39–11.17), and 8.9 (95% CI: 6.31–11.56), respectively (Figure 2B and Supplementary Table 2).

### Age and sex differences in the burden of LOC-HAC

**Figure 3:**
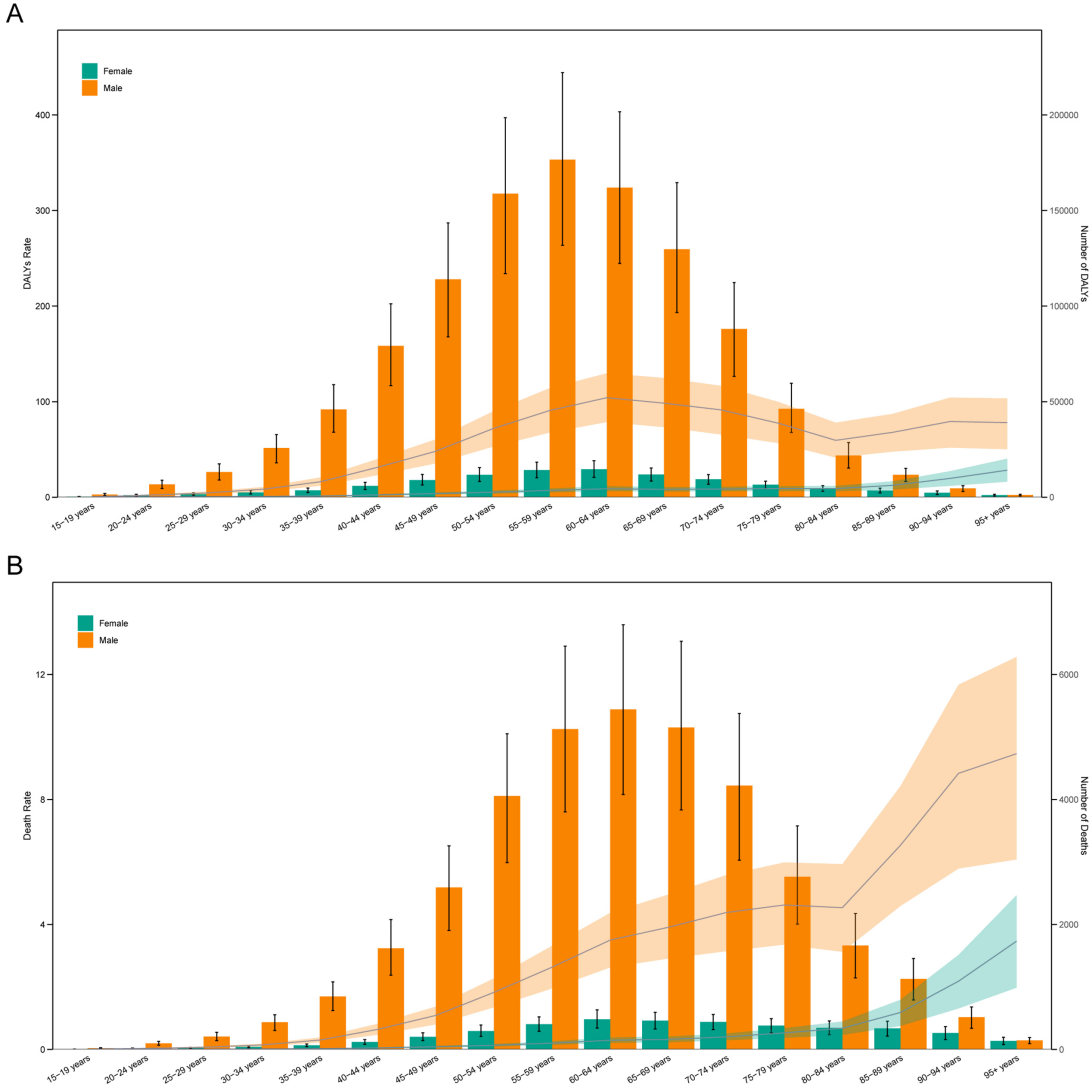
Age-specific numbers and rates of DALYs **(A)** and deaths **(B)** of lip and oral cavity cancer attributable to high alcohol consumption by age and sex in 2021.

In 2021, DALYs and death cases in males were significantly higher than in females, and both increased with age. DALYs cases in males peaked in the 55–59 age group, while in females they peaked in the 60–64 age group. For both sexes, death cases peaked in the 60–64 age group. DALYs rates and death rates in males were higher than in females. The highest DALYs rate in males was in the 60–64 age group, whereas in females it was in the 95 + age group. For males and females, the death rate was highest in the 95 + age group (Figures 3A,B).

### Relationship between the burden of LOC-HAC and SDI

**Figure 4:**
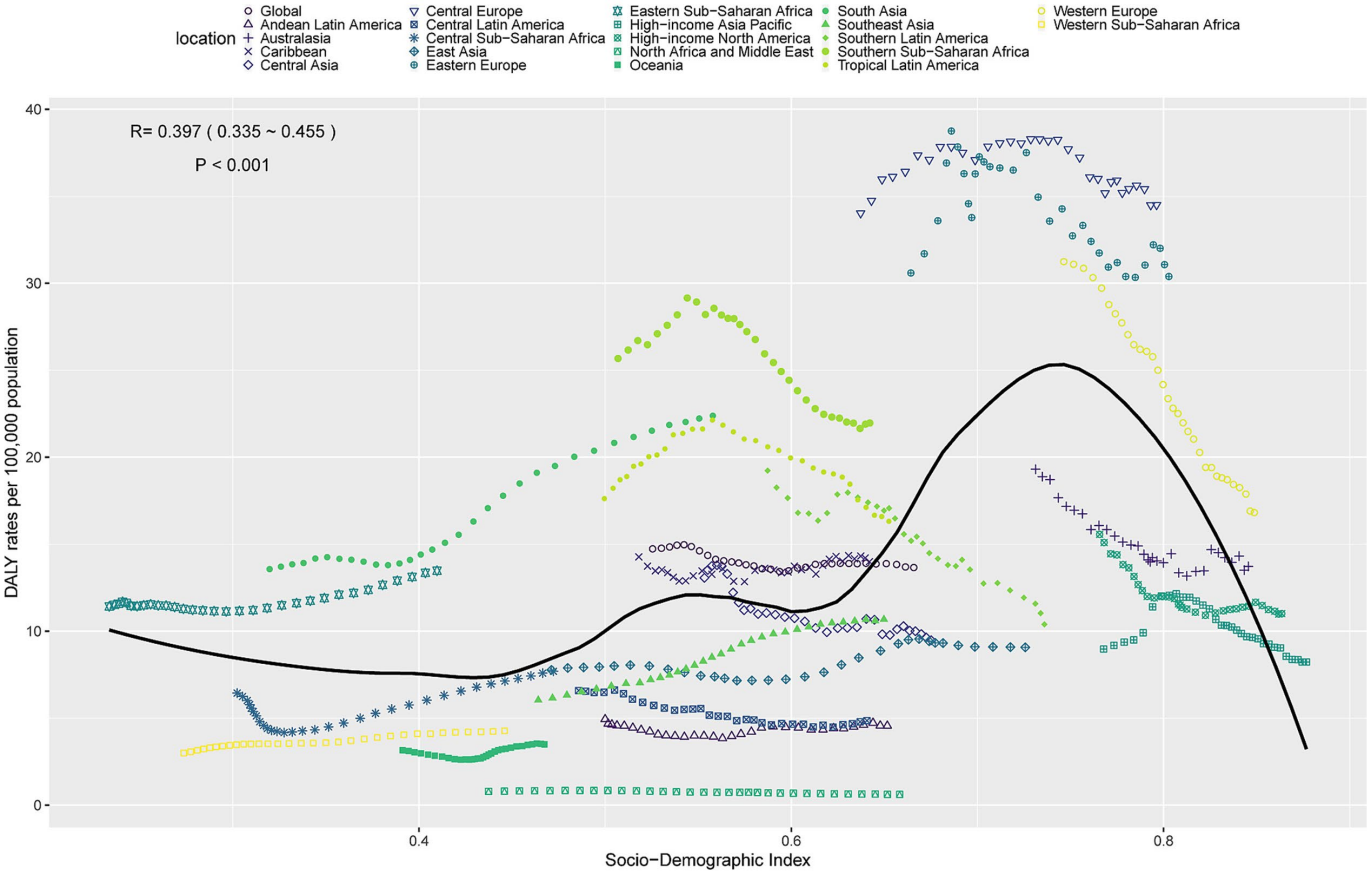
ASDR of lip and oral cavity cancer attributable to high alcohol consumption in 21 GBD regions by SDI, 1990–2021.

In 2021, ASDR and ASMR of LOC-HAC showed a significant positive correlation with SDI, indicating that the disease burden increased with higher SDI levels (Figure 4 and Supplementary Figure 2). Specifically, when SDI ranged from 0.6 to 0.75, ASDR and ASMR rose rapidly with increasing SDI. However, when SDI exceeded 0.75, ASDR and ASMR decreased sharply. Notably, regions including Eastern Sub-Saharan Africa, South Asia, Central Europe, Western Europe, and Eastern Europe had burdens significantly higher than expected, whereas North Africa and Middle East, Oceania, Andean Latin America, East Asia, and Southeast Asia had burdens significantly lower than expected, warranting further investigation.

### Decomposition analysis of the burden of LOC-HAC

**Figure 5:**
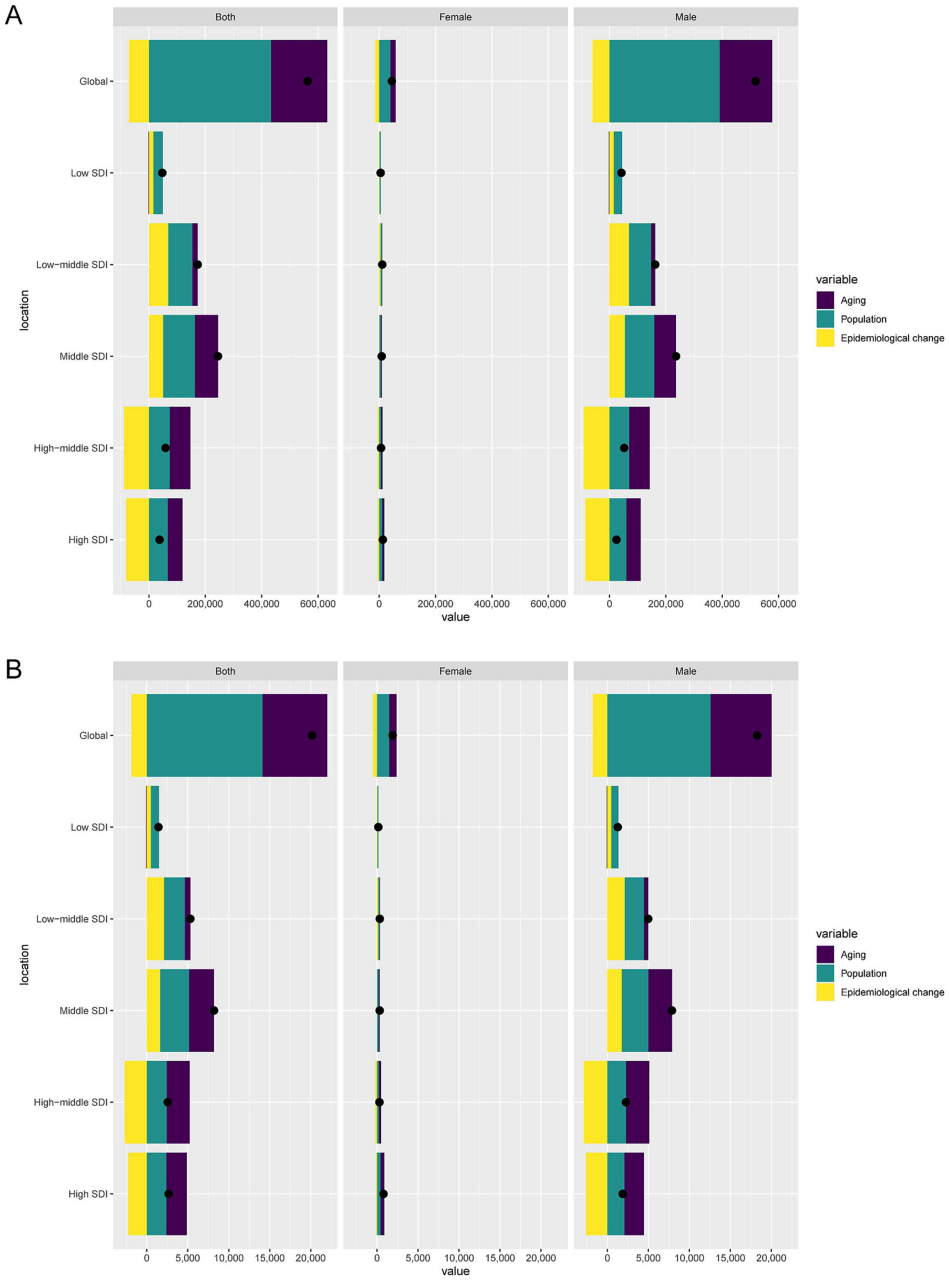
Decomposition analysis of changes in DALYs **(A)** and deaths **(B)** of lip and oral cavity cancer attributable to high alcohol consumption between 1990 and 2021 across SDI regions.

Decomposition analysis showed that over the past 32 years, global DALYs cases increased by 562,600.36, of which aging contributed 200,222.09 (35.59%), population growth contributed 432,682.39 (76.91%), and epidemiological change contributed −70,304.13 (−12.5%). Among males, DALYs cases increased by 517,802.3, with aging contributing 186,677.18 (36.05%), population growth contributing 390,698.27 (75.45%), and epidemiological change contributing −59,573.14 (−11.5%). Among females, DALYs cases increased by 44,798.05, with aging contributing 18,886.41 (42.16%), population growth contributing 39,500.81 (88.18%), and epidemiological change contributing −13,589.17 (−30.33%) (Figure 5A).

Globally, death cases increased by 20,174.61, with aging contributing 7,902.96 (39.17%), population growth contributing 14,121.34 (70%), and epidemiological change contributing −1,849.7 (−9.17%). Among males, death cases increased by 18,276.3, with aging contributing 7,441.35 (40.72%), population growth contributing 12,581.82 (68.84%), and epidemiological change contributing −1,746.87 (−9.56%). Among females, death cases increased by 1,898.31, with aging contributing 917.42 (48.33%), population growth contributing 1,470.21 (77.45%), and epidemiological change contributing −489.33 (−25.78%) (Figure 5B).

### Cross-country inequality analysis of the burden of LOC-HAC

**Figure 6:**
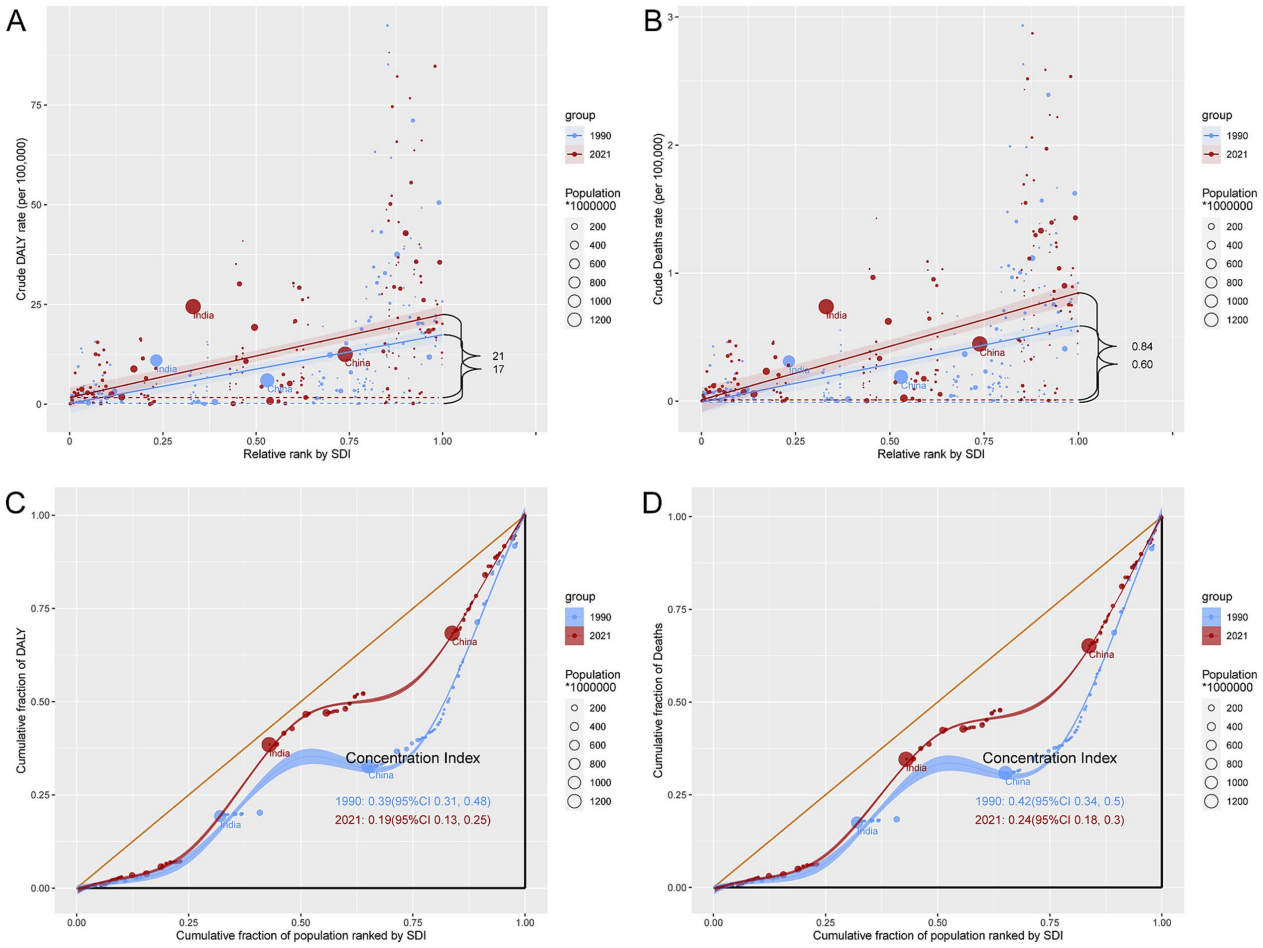
Inequality analysis of DALYs and mortality in lip and oral cavity cancer attributable to high alcohol consumption in 1990 and 2021 across the world. **(A)** Health inequality regression curves for DALYs. **(B)** Health inequality regression curves for mortality. **(C)** Concentration curves for DALYs. **(D)** Concentration curves for mortality.

Between 1990 and 2021, there were significant health inequalities in LOC-HAC burden across countries with different development levels. Specifically, the SII for DALYs rates increased from 17 in 1990 to 21 in 2021, indicating a widening gap in DALYs rates between the highest-SDI and lowest-SDI regions. The SII for death rates increased from 0.60 in 1990 to 0.84 in 2021. Meanwhile, the CI for DALYs rates decreased from 0.39 in 1990 to 0.19 in 2021, and the CI for death rates decreased from 0.42 to 0.24, suggesting that relative inequality was alleviated. However, higher-SDI regions still bore a heavier disease burden (Figures 6A-D).

### Forecast analysis of the burden of LOC-HAC

**Figure 7:**
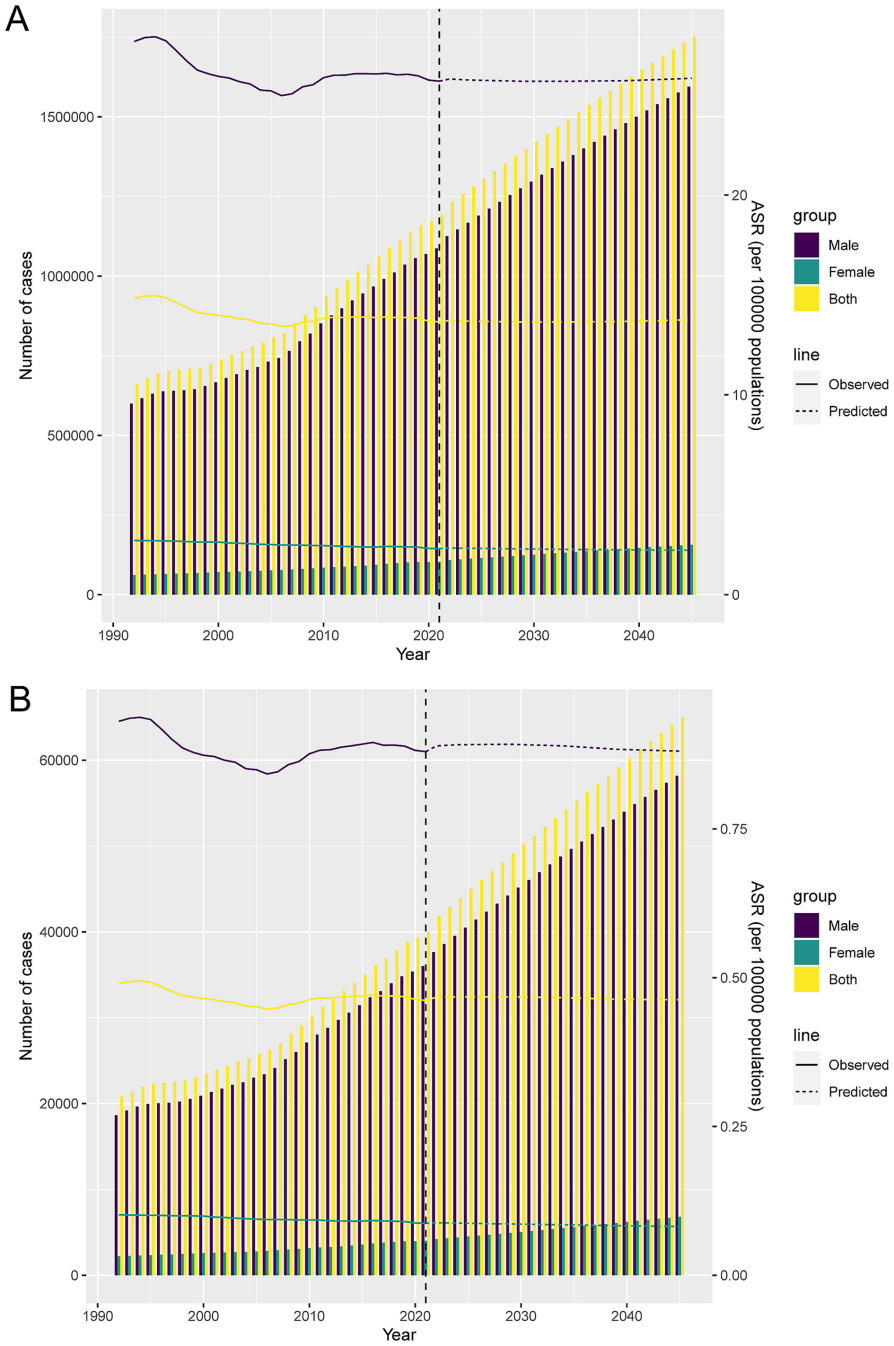
Projections of the temporal trends of the number of DALYs cases, mortality cases, ASDR, and ASMR of lip and oral cavity cancer attributable to high alcohol consumption globally up to 2045. **(A)** The number and ASDR of lip and oral cavity cancer attributable to high alcohol consumption by year and gender. **(B)** The number and ASMR of lip and oral cavity cancer attributable to high alcohol consumption by year and gender.

Forecast analysis suggests that from 2022 to 2045, DALYs and death cases in males will rise significantly, while those in females will increase slowly. Meanwhile, the DALY rates and death rates for males and females will remain relatively stable, with males having much higher DALY rates and death rates than females (Figures 7A,B).

## Discussion

The global burden of LOC-HAC has increased significantly over the past few decades, especially in regions with a low to middle SDI and Southeast Asia. In 2021, the number of DALY cases globally had risen by 90% compared to 1990, with the fastest growth occurring in low to middle SDI regions, where the percentage change was 279%. The number of death cases also showed a similar trend, with a 296% increase in low-middle SDI regions. Among the 21 GBD regions, Southeast Asia saw the most significant rise in DALY and death cases, with increases of 326 and 357%, respectively. This may be related to local lifestyle factors, such as betel nut chewing, and inadequate allocation of medical resources (29).

Although global ASDR and ASMR have declined overall, rapid increases in Southeast Asia highlight the need for strengthened health education and medical interventions. Central Europe had the highest ASDR and ASMR, indicating ongoing challenges. In the future, low-middle SDI and Southeast Asia should be prioritized, with measures such as lifestyle improvement, early screening, and optimized healthcare resource allocation to reduce the incidence and mortality of LOC-HAC (30).

From 1990 to 2021, LOC-HAC burden rose markedly in several countries, with Iran, Libya, Cape Verde, Viet Nam, and Nepal experiencing the fastest increases in DALYs and death cases. These findings suggest that these countries may face high-risk behaviors (e.g., smoking, alcohol use) and limited healthcare resources. In Iran, major health system reforms and improved reporting may have contributed to the substantial increases. Some data may also have been influenced by exceptional events such as policy changes or targeted health interventions.

In 2021, DALYs and death cases in males were significantly higher than in females, and both increased with age. Male DALYs peaked at 55–59 years, while female DALYs peaked at 60–64 years. These differences may be due to variations in survival, exposure levels, and diagnostic practices between sexes. Men may be more likely to engage in high-risk behaviors earlier, while women may experience later exposures. Additionally, differences in diagnosis may account for the variation in peak ages.

Although SDI was positively correlated with ASDR and ASMR, both declined when SDI exceeded 0.75. This may be explained by better healthcare access, preventive strategies, and changing alcohol consumption patterns in high-SDI regions. Comprehensive health education, early detection, and effective public health policies in these regions may have reduced the burden of LOC-HAC.

Over the past 32 years, the global disease burden of LOC-HAC has increased significantly, driven mainly by population aging and population growth, while epidemiological changes have had a certain mitigating effect. In addition, global health inequality has become more pronounced between countries at different levels of socio-economic development, with the gap in disease burden between high SDI regions and low SDI regions gradually widening, and high SDI regions still bearing a heavier burden. This means that the improvement in health status in high SDI regions may be faster than that in low SDI regions, or the deterioration in health status in low SDI regions may be more rapid. Therefore, it suggests that policymakers need to pay more attention to low SDI regions in terms of resource allocation and health interventions to narrow the health gap.

Assuming current alcohol consumption trends and policies remain unchanged, forecasts suggest that from 2022 to 2045, DALYs and death cases among males will rise substantially, while increases among females will be modest. ASDR and ASMR are expected to remain relatively stable but consistently higher in males. This underscores the need for interventions targeting high-risk behaviors in men while continuing to address women’s health needs.

Extensive evidence has established alcohol and its metabolite acetaldehyde as Group 1 carcinogens, acting through multiple mechanisms (31). Ethanol metabolism produces acetaldehyde, which damages DNA and impairs repair pathways, and generates ROS that drive oxidative stress and genomic instability (32). Alcohol also alters hormone levels, suppresses immune surveillance, and enhances the carcinogenicity of tobacco and other agents (33). Together, these pathways markedly increase the risk of several cancers, including oral cancer.

Several policy interventions have proven effective in substantially reducing alcohol consumption (34). For instance, increasing taxation on alcoholic beverages has been shown to decrease consumption, with particularly strong effects observed among low-income populations and younger individuals. Similarly, the implementation of minimum unit pricing policies can discourage the excessive intake of inexpensive alcoholic products, thereby mitigating harmful drinking behaviors (34, 35). Moreover, restricting the hours and days of alcohol sales and limiting outlet density have demonstrated efficacy in reducing alcohol availability, leading to lower consumption and associated health risks (36). Given the considerable disease burden of LOC, a multifaceted approach is warranted. In low-middle SDI regions and in Southeast Asia, health education campaigns should be prioritized to improve public awareness of high-risk behaviors, including tobacco use, alcohol consumption, and betel quid chewing, and to promote healthier lifestyle choices. The establishment of early screening programs for LOC is also crucial, particularly in high-incidence countries and among older populations, to enhance early detection and improve treatment outcomes. In parallel, optimizing the allocation of healthcare resources by expanding treatment facilities and increasing the availability of trained specialists in resource-limited settings will be essential. Furthermore, the development and enforcement of alcohol- and tobacco-control policies can reduce the prevalence of high-risk behaviors, with tailored interventions targeting men, who remain disproportionately affected. Finally, strengthening international collaboration to share best practices and coordinate responses will be vital, with a focus on regions and populations carrying the heaviest burden, in order to achieve meaningful reductions in the incidence and mortality of LOC.

This study has some limitations. First, GBD data quality and representativeness in low- and middle-income countries may be limited due to less robust data collection and reporting systems. Second, while this study systematically analyzed epidemiological changes across regions and countries, it did not explore subpopulations (e.g., provincial, urban–rural differences). Third, LOC-HAC may suffer from underreporting or misclassification. Fourth, the COVID-19 pandemic may have affected data collection and reporting for 2021, leading to bias or incompleteness.

## Conclusion

Based on GBD 2021 data, this study analyzed global trends in LOC-HAC burden from 1990 to 2021. The results show significant increases in DALYs and death cases, with the greatest rises in low-middle SDI regions and Southeast Asia. Males had higher DALYs cases than females, with peaks at ages 55–59 for males and 60–64 for females. Substantial global health inequalities were observed, with widening absolute differences between the highest and lowest SDI regions. Forecasts suggest that from 2022 to 2045, DALYs and death cases will increase significantly in males, while females will show slower increases. This study recommends prioritizing low-middle SDI and Southeast Asia by promoting healthy lifestyles, strengthening early screening, and optimizing healthcare resource allocation to reduce the incidence and mortality of LOC-HAC.

## Data Availability

The original contributions presented in the study are included in the article/Supplementary material, further inquiries can be directed to the corresponding author/s.
