# Supplementary material for: Global burden of lip and oral cavity cancer attributable to high alcohol consumption from 1990 to 2021
Source: Front Nutr. 2025 Sep 29;12:1648788. doi: 10.3389/fnut.2025.1648788 (PMC12515677; doi:10.3389/fnut.2025.1648788)
Supplement: Supplementary file 1 [file Image_1.pdf]

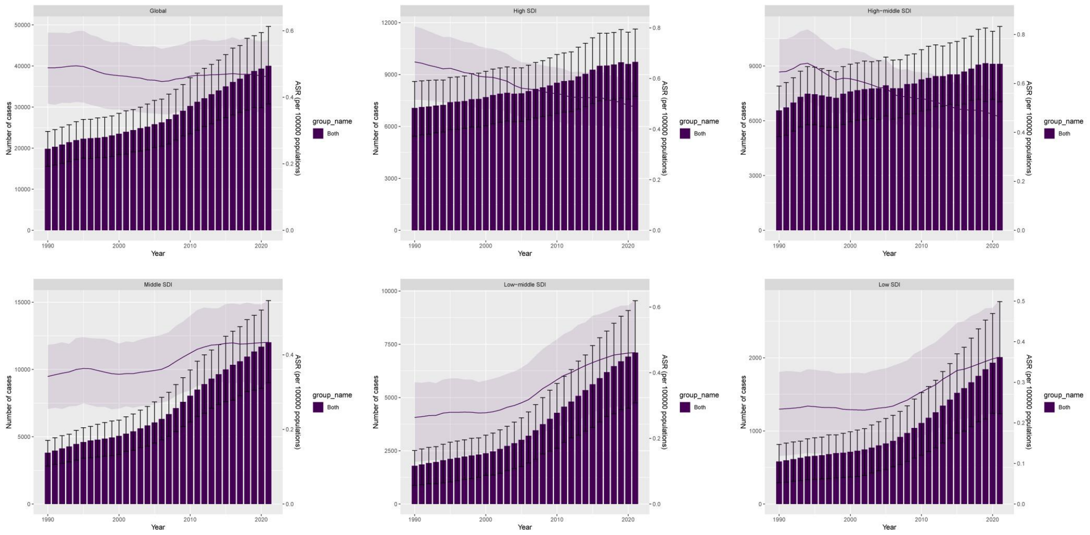

Supplementary figure 1. Death cases and ASMR of lip and oral cavity cancer attributable to high alcohol consumption from 1990 to 2021.

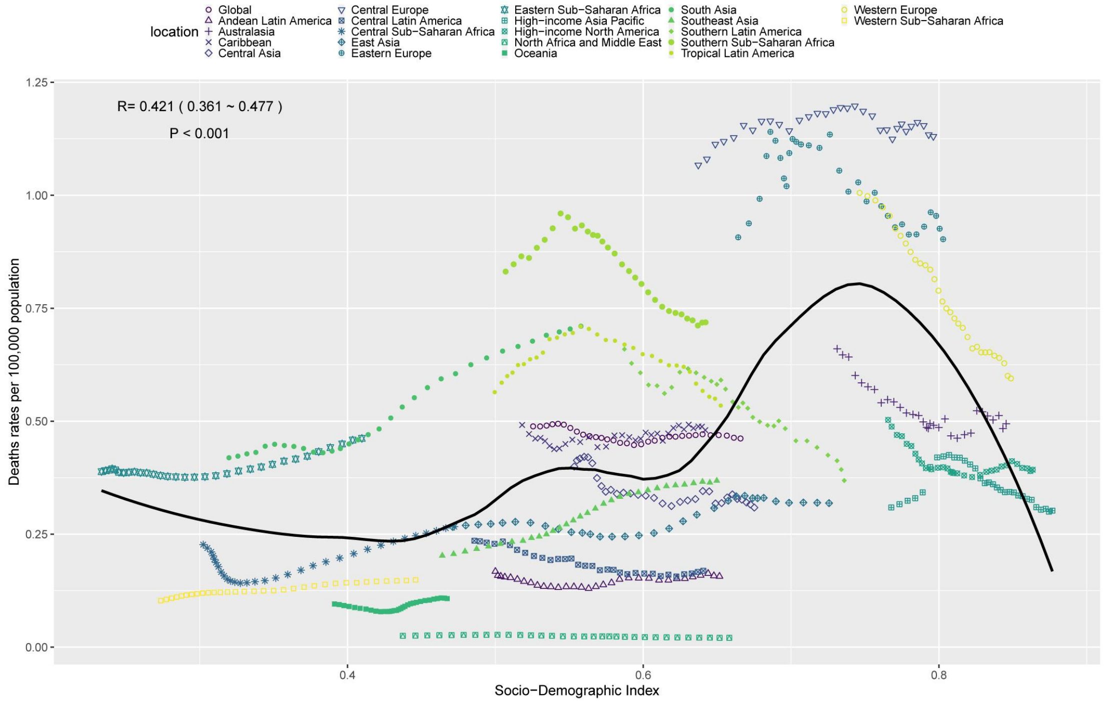

Supplementary figure 2. ASMR of lip and oral cavity cancer attributable to high alcohol consumption in 21 GBD regions by SDI, 1990–2021.
