# Supplementary material for: Global burden of lip and oral cavity cancer attributable to high alcohol consumption from 1990 to 2021
Source: Front Nutr. 2025 Sep 29;12:1648788. doi: 10.3389/fnut.2025.1648788 (PMC12515677; doi:10.3389/fnut.2025.1648788)
Supplement: Supplementary file 2 [file Table_1.pdf]

Location	1990_DALYs cases (95% UI)	2021_DALYs cases (95% UI)	Percentage change	1990_ASDR_per 100000(95% UI)	2021_ASDR_per 100 000(95% UI)	EAPC (95% CI)
Albania	121.68 (66.14-191.34)	366.15 (221.71-584.04)	2.01	5.17 (2.72-8.17)	9.19 (5.51-14.57)	2.09 (1.43-2.74)
Algeria	87.48 (48.14-131.66)	314.94 (178.55-499.39)	2.6	0.6 (0.31-0.91)	0.76 (0.43-1.21)	0.97 (0.84-1.09)
American Samoa	0.05 (0.01-0.17)	0.11 (0.02-0.29)	1.2	0.17 (0.02-0.52)	0.21 (0.03-0.55)	1.51 (0.51-2.52)
Andorra	9.89 (6.38-14.59)	14.39 (8.55-21.22)	0.46	16.69 (10.74-24.56)	9.64 (5.76-14.11)	-1.57 (-1.73--1.4)
Angola	327.78 (130.81-562.66)	1957.31 (1135.5-2873.74)	4.97	6.45 (2.59-11.04)	12.58 (7.44-18.25)	2.89 (2.44-3.34)
Antigua and Barbuda	3.16 (1.47-4.96)	11.76 (8.64-15.1)	2.72	6.51 (3.05-10.28)	10.33 (7.62-13.22)	1.51 (1.22-1.8)
Argentina	6921.02 (5549.61-8415.69)	6019.61 (4497.15-7504.92)	-0.13	21.31 (17.13-25.91)	11.32 (8.48-14.07)	-1.58 (-1.86--1.31)
Armenia	154.21 (98.91-220.65)	277.31 (185.29-375.4)	0.8	4.9 (3.11-7.07)	6.65 (4.47-8.95)	1.37 (0.96-1.77)
Australia	3669.65 (2649.37-4631.97)	5565.14 (4318.86-6911.65)	0.52	19.46 (14.2-24.47)	13.93 (10.79-17.25)	-0.99 (-1.19--0.79)
Austria	3044.86 (2421.69-3589.28)	2740.36 (2158.83-3356.39)	-0.1	30.52 (24.52-35.95)	17.84 (14.18-21.8)	-1.74 (-1.87--1.61)
Azerbaijan	269.87 (155.73-436.62)	504.2 (250.2-846.77)	0.87	4.69 (2.7-7.63)	4.15 (2.11-6.82)	0.13 (-0.15-0.4)
Bahrain	8.02 (5.35-11.48)	17.52 (9.4-26.35)	1.18	2.85 (1.84-4.08)	1.2 (0.63-1.81)	-3.05 (-3.36--2.72)
Bangladesh	332.79 (0-1142.57)	4083.92 (178.12-9466.59)	11.27	0.57 (0-1.96)	2.65 (0.11-6.13)	4.94 (4.37-5.51)

Barbados	38.55 (28.6-48.28)	66.15 (47.23-93.9)	0.72	15.42 (11.48-19.37)	13.67 (9.75-19.32)	0.02 (-0.13-0.17)
Belarus	6604.21 (4819.59-8249.9)	6134.42 (4233.12-8334.56)	-0.07	51.49 (37.91-64.17)	42.13 (29.39-56.83)	-0.86 (-1.05--0.68)
Belgium	3549.87 (2821.27-4260.65)	3679.46 (2904.23-4525.35)	0.04	26.77 (21.57-31.85)	19.3 (15.3-23.62)	-1.25 (-1.51--0.99)
Belize	6.13 (4.5-7.71)	21.74 (15.61-28.21)	2.55	6.23 (4.57-7.87)	6.21 (4.43-8.1)	0.51 (0.1-0.92)
Benin	38.47 (16.4-63.38)	208.46 (105.35-343.83)	4.42	1.74 (0.73-2.9)	3.2 (1.59-5.31)	2 (1.85-2.15)
Bermuda	23.52 (17.91-28.75)	20.69 (14.78-27.29)	-0.12	36.49 (27.73-44.68)	17.19 (12.43-22.61)	-1.99 (-2.36--1.63)
Bhutan	81.71 (36.9-130.25)	25.5 (6.26-56.81)	-0.69	25.27 (11.13-40.44)	3.63 (0.91-8.15)	-7.9 (-8.52--7.28)
Bolivia	240.53 (127.19-376.8)	613.66 (388.55-929.09)	1.55	6.42 (3.36-10.14)	6.05 (3.82-9.24)	0.01 (-0.21-0.23)
Bosnia and Herzegovina	564.92 (378.64-760.27)	911.74 (643.71-1220.73)	0.61	11.83 (7.92-15.94)	16.33 (11.56-22.23)	1.27 (1.13-1.4)
Botswana	124.24 (65.94-197.53)	304.8 (158.99-481.17)	1.45	18.27 (9.68-28.76)	16.07 (8.48-24.93)	-0.91 (-1.23--0.59)
Brazil	18326.26 (14481.9-22520.81)	42427.86 (32985.34-52668.36)	1.32	17.71 (14.01-21.8)	16.33 (12.68-20.26)	-0.4 (-0.7--0.1)
Brunei	7.53 (5.11-10.99)	4.73 (0.39-9.69)	-0.37	4.96 (3.22-7.22)	1 (0.08-2.05)	-1.5 (-3.43-0.46)
Bulgaria	2387.05 (1836.02-2985.07)	3544.82 (2603.24-4610.52)	0.49	20.38 (15.66-25.74)	31.3 (22.89-40.58)	0.7 (0.38-1.02)
Burkina Faso	215.43 (124.51-307.12)	674.01 (413.88-955.13)	2.13	4.43 (2.59-6.25)	6.22 (3.9-8.8)	0.86 (0.66-1.07)
Burundi	774.8 (437.5-1111.31)	1035.72 (608.18-1585.2)	0.34	28.99 (16.91-41.	16.42 (9.45-25.0	-2.35 (-2.53--2.1

				52)	4)	6)
Cambodia	177.99 (74.82-330.57)	2318.65 (1513.23-3450.48)	12.03	3.26 (1.37-6.08)	16.25 (10.69-24.14)	4.85 (4.51-5.19)
Cameroon	274.09 (146.16-402.42)	1420.91 (898.5-2098.68)	4.18	5.14 (2.78-7.58)	8.94 (5.7-13.07)	2.09 (1.96-2.23)
Canada	5509.17 (3849-7229.29)	7016.53 (5111.39-9158.49)	0.27	17.69 (12.43-23.16)	11.1 (8.18-14.32)	-1.13 (-1.3--0.96)
Cape Verde	1.5 (0.86-2.14)	105.83 (65.01-167.01)	69.55	0.75 (0.43-1.07)	20.91 (13.04-32.66)	9.05 (6.45-11.71)
Central African Republic	124.81 (50.07-249.26)	150.9 (56.68-307.07)	0.21	8.84 (3.78-17.34)	5.04 (1.9-10.12)	-1.68 (-1.76--1.59)
Chad	25.77 (2.41-69.84)	298.55 (81.22-581.58)	10.59	0.83 (0.08-2.26)	4.09 (1.07-7.92)	6.33 (5.8-6.86)
Chile	1168.1 (944.01-1420.27)	1683.6 (1315.37-2126.67)	0.44	10.91 (8.82-13.31)	6.79 (5.32-8.58)	-1.25 (-1.38--1.12)
China	70406.16 (50590.82-89463.35)	178068.27 (125053.16-242770.64)	1.53	7.41 (5.32-9.46)	8.32 (5.9-11.31)	0.71 (0.35-1.08)
Colombia	1639.79 (1273-2071.78)	2145.68 (1428.47-2975.74)	0.31	7.76 (5.99-9.9)	3.89 (2.58-5.41)	-2.88 (-3.22--2.54)
Comoros	1.89 (0.15-5.23)	11.53 (3.51-23.14)	5.1	0.77 (0.06-2.15)	2 (0.6-4.02)	3.52 (3.28-3.76)
Congo	105.2 (37.16-196.59)	476.4 (252.44-712.1)	3.53	8.44 (3.04-15.62)	13.81 (7.54-20.45)	2.44 (1.76-3.12)
Cook Islands	0.23 (0-0.71)	2.92 (1.93-4.06)	11.7	1.56 (0-4.9)	12.38 (8.18-17.22)	8.31 (7.3-9.32)
Costa Rica	189.46 (146.84-234.19)	320.11 (230.7-414.99)	0.69	10.05 (7.74-12.54)	5.8 (4.18-7.53)	-2.42 (-2.77--2.08)
Cote d'Ivoire	455.63 (227.78-703.66)	1842.4 (951.26-2957.09)	3.04	8.61 (4.18-13.29)	12.42 (6.64-19.32)	0.88 (0.71-1.05)
Croatia	3003.17	1920.44	-0.36	46.82	25.91	-1.98

	(2273.16-3655.1)	(1471.25-2404.05)		(35.88-56.93)	(19.84-32.6)	(-2.12--1.85)
Cuba	1475 (1051.58-1911.61)	3418.69 (2427.42-4466.35)	1.32	14.29 (10.14-18.55)	18.22 (12.92-23.78)	1.18 (1-1.35)
Cyprus	92.03 (68.03-123.66)	160.03 (112.61-215.65)	0.74	11.82 (8.67-15.77)	8.27 (5.82-11.17)	-0.88 (-1.04--0.73)
Czech Republic	4808.88 (3822.8-5801.11)	5122.98 (4021.8-6330.96)	0.07	37.53 (29.9-45.2)	28.6 (22.25-35.53)	-0.91 (-1.02--0.81)
Democratic People's Republic of Korea	1356.12 (830.18-2007.36)	2372.24 (1413.3-3672.32)	0.75	7.02 (4.35-10.34)	6.81 (4.07-10.5)	0.07 (-0.07-0.21)
Democratic Republic of the Congo	1058.34 (370.57-1800.59)	2555.68 (1002.96-4311.77)	1.41	5.66 (2.07-9.6)	5.46 (2.19-9.28)	0.18 (-1.49-1.87)
Denmark	1149.26 (905.28-1369.33)	1464.53 (1152.74-1793.92)	0.27	16.74 (13.33-19.93)	14.48 (11.61-17.68)	-1.17 (-1.77--0.57)
Djibouti	9.88 (2.09-20.2)	5.73 (1.07-14.98)	-0.42	4.97 (1.24-10.12)	0.6 (0.12-1.51)	-8.14 (-8.87--7.39)
Dominica	10.86 (8.03-14.37)	15.89 (10.38-22.11)	0.46	19 (14.05-25.1)	18.44 (12.08-25.59)	0.07 (-0.04-0.17)
Dominican Republic	478.41 (337.72-659.56)	1314.49 (814.87-2038.98)	1.75	11.03 (7.77-15.33)	12.48 (7.63-19.41)	0.44 (0.26-0.62)
Ecuador	183.55 (116.58-246.31)	554.55 (374.61-784.1)	2.02	2.88 (1.79-3.89)	3.23 (2.18-4.56)	1.28 (0.85-1.7)
Egypt	26.66 (14.51-40.73)	137.13 (79.4-202.72)	4.14	0.07 (0.04-0.11)	0.16 (0.09-0.24)	3.98 (3.27-4.7)
El Salvador	158.27 (112.66-202.86)	277.09 (193.15-387.82)	0.75	4.8 (3.38-6.2)	4.55 (3.16-6.4)	-0.67 (-0.94--0.4)
Equatorial Guinea	14.42 (4.65-28.88)	88.79 (48.3-137.61)	5.16	6.26 (2.02-12.43)	13.27 (7.43-20.01)	2.66 (2.39-2.93)
Eritrea	100.95 (24.3-196.44)	158.88 (49.08-299)	0.57	5.89 (1.43-11.37)	3.89 (1.26-7.27)	-2.96 (-3.47--2.44)
Estonia	457.35	525.1	0.15	23.25	24.88	-0.33

	(328.19-600.55)	(366.15-690.09)		(17.07-30.38)	(17.93-32.19)	(-0.66-0)
Ethiopia	992.07 (286.91-2151.59)	4078.8 (2354.75-6257.4)	3.11	4.06 (1.24-8.78)	7.79 (4.31-12.18)	2.26 (1.54-2.98)
Fiji	23.09 (11.81-34.86)	53.07 (28.66-79.38)	1.3	4.48 (2.23-6.83)	5.88 (3.15-8.79)	1.22 (0.81-1.64)
Finland	894.95 (713.06-1078.97)	995.16 (768.56-1219.67)	0.11	13.88 (11.2-16.73)	10.48 (8.24-12.74)	-0.68 (-0.83--0.53)
France	41087.62 (32450.06-49367.43)	23709.69 (18857.11-28689.48)	-0.42	58.1 (46.3-69.87)	21.64 (17.18-26.33)	-3.28 (-3.53--3.04)
Gabon	114.35 (63.39-172.57)	209.03 (120.6-293.43)	0.83	18.86 (10.62-28.44)	16.89 (9.86-23.55)	-0.54 (-0.62--0.45)
Georgia	741.74 (450.18-1068.58)	1052.84 (702.63-1381.8)	0.42	11.62 (7.05-16.63)	19.67 (13.58-25.62)	2.96 (2.2-3.73)
Germany	40383.7 (32466.67-47686.21)	30367.62 (24415.75-36446.58)	-0.25	36.68 (29.61-43.13)	19.2 (15.6-23.01)	-2.06 (-2.21--1.92)
Ghana	111.39 (54.5-176.27)	226.81 (113-359.22)	1.04	1.43 (0.71-2.26)	1.08 (0.54-1.73)	-1.9 (-2.4--1.4)
Greece	1574.79 (1231.73-1890.89)	2203.45 (1707.43-2685.51)	0.4	11.1 (8.82-13.27)	12.14 (9.45-14.79)	0.11 (-0.03-0.25)
Greenland	14.93 (8.98-21.13)	18.92 (11.6-25.91)	0.27	35.88 (20.85-52.49)	23.72 (14.54-32.49)	-1.15 (-1.28--1.02)
Grenada	13.58 (9.96-17.42)	20.92 (14.41-27.21)	0.54	21.45 (15.79-27.54)	17.01 (11.81-22.09)	-0.59 (-0.82--0.37)
Guam	2.04 (0.03-5.06)	19.2 (4.43-33.15)	8.41	1.91 (0.02-4.83)	10.12 (2.42-17.4)	7.25 (6.67-7.84)
Guatemala	197.52 (145.85-251.38)	327.61 (228.14-460.92)	0.66	4.68 (3.43-6.08)	2.67 (1.85-3.79)	-2.29 (-2.51--2.06)
Guinea	122.41 (41.44-230.49)	490.38 (209.9-842.27)	3.01	3.41 (1.13-6.42)	7.34 (3.08-12.69)	1.87 (1.15-2.59)

Guinea-Bissau	20.46 (9.64-34.98)	57.18 (33.27-92.07)	1.79	4.34 (2.08-7.42)	5.85 (3.37-9.38)	0.88 (0.63-1.14)
Guyana	57.43 (42.61-73.17)	71.23 (47.95-100.64)	0.24	13.17 (9.72-16.85)	9.68 (6.51-13.68)	-0.36 (-0.55--0.17)
Haiti	630.53 (392.72-1089.8)	1272.78 (783.38-1946.01)	1.02	16.89 (10.51-28.92)	14.8 (9.05-22.61)	-0.31 (-0.36--0.26)
Honduras	73.87 (48.23-99.97)	257.73 (168.32-386.08)	2.49	3.12 (2.07-4.24)	3.66 (2.35-5.58)	0.38 (0.25-0.5)
Hungary	9868.15 (7678.03-12052.43)	7883.09 (6019.27-10274.69)	-0.2	73.54 (57.96-89.55)	49.27 (37.66-64.04)	-1.89 (-2.3--1.48)
Iceland	25.4 (17.35-33.92)	58.7 (44.68-73.76)	1.31	9.81 (6.7-13.1)	11.62 (8.93-14.58)	0.65 (0.54-0.75)
India	93651.36 (42488.48-132870.57)	345984.1 (233416.26-469124.92)	2.69	16.02 (7.14-22.61)	25.83 (17.35-35.09)	1.85 (1.6-2.09)
Indonesia	987.49 (306.05-1758.02)	2216.43 (609.46-4449.67)	1.24	0.78 (0.24-1.4)	0.76 (0.21-1.5)	-0.68 (-1.01--0.36)
Iran	0.02 (0-0.11)	307.24 (199.37-435.78)	15361	0 (0-0)	0.34 (0.22-0.48)	38.43 (31.22-46.04)
Iraq	37.46 (23.18-54.55)	75.2 (44.86-115.58)	1.01	0.39 (0.24-0.58)	0.24 (0.14-0.37)	-1.81 (-1.98--1.65)
Ireland	891.73 (699.91-1098.18)	931.6 (724.88-1137.33)	0.04	23.58 (18.74-28.96)	13.03 (10.21-15.87)	-1.96 (-2.12--1.8)
Israel	85.16 (41.95-134.38)	324.56 (206.42-451.91)	2.81	1.9 (0.94-2.98)	2.99 (1.88-4.14)	1.82 (1.54-2.11)
Italy	24238.45 (19381.66-28717.29)	17326.17 (13662.71-20865.94)	-0.29	30.05 (24.24-35.51)	14.56 (11.65-17.5)	-2.5 (-2.7--2.31)
Jamaica	87.9 (58.95-118.43)	164.07 (104.64-244.14)	0.87	5.23 (3.5-7.03)	5.32 (3.39-7.91)	0.13 (-0.38-0.64)
Japan	14864.37 (11933.73-1765	23396.63 (17424.76-28720.	0.57	8.89 (7.16-10.5	9 (7.07-10.8	-0.46 (-0.89--0.0

	7.61)	81)		6)	4)	4)
Jordan	7.59 (4.47-11.61)	38.21 (21.65-59.3)	4.03	0.44 (0.25-0.68)	0.39 (0.22-0.62)	-0.18 (-0.62-0.27)
Kazakhstan	3579.71 (2501.3-4522.45)	2875.87 (1945.03-3777.07)	-0.2	25.15 (17.4-32)	14.25 (9.64-18.73)	-2.51 (-2.74--2.28)
Kenya	1672.96 (785.49-2456.13)	5740.26 (3555.44-8035.29)	2.43	17.17 (8.28-25.15)	19.93 (12.51-27.62)	0.68 (0.51-0.85)
Kiribati	5.71 (1.03-11.37)	6.49 (0.67-16.81)	0.14	11.69 (1.92-23.57)	6.62 (0.65-17.13)	-2.35 (-2.74--1.96)
Kyrgyzstan	928.02 (632.97-1196.42)	517.38 (369.31-698.82)	-0.44	28.91 (19.74-37.26)	8.78 (6.26-11.93)	-2.27 (-3.04--1.49)
Lao People's Democratic Republic	241.63 (116.69-412.58)	773.27 (491.69-1089.64)	2.2	9.82 (4.72-16.74)	13.84 (8.74-19.45)	1.38 (0.98-1.78)
Latvia	1034.89 (768.08-1314.31)	1189.77 (859.54-1560.52)	0.15	30.26 (22.46-38.19)	38.34 (28.18-50.1)	0.46 (0.23-0.68)
Lebanon	93.58 (51.78-162.46)	120.9 (71.69-187.89)	0.29	3.84 (2.11-6.66)	2.07 (1.21-3.23)	-2.01 (-2.12--1.91)
Lesotho	123.02 (66.99-190.57)	297.09 (142.2-518.19)	1.41	13.17 (7.12-20.47)	23.44 (11.18-41.11)	2.09 (1.85-2.33)
Liberia	58.83 (33.37-89.71)	155.98 (89.88-241.37)	1.65	4.69 (2.69-7.17)	5.16 (2.98-8.21)	0.08 (-0.05-0.21)
Libya	0.13 (0.03-0.34)	39.76 (22.75-65.04)	304.85	0 (0-0.01)	0.56 (0.31-0.91)	11.54 (7.48-15.75)
Lithuania	1182.29 (862.76-1481.57)	1803.65 (1301.9-2283.06)	0.53	26.98 (19.79-33.7)	40.11 (29.43-50.54)	0.82 (0.55-1.08)
Luxembourg	187.63 (153.06-223.65)	167.24 (131.32-204.39)	-0.11	37.32 (30.42-44.49)	16.83 (13.29-20.56)	-2.45 (-2.56--2.34)
Macedonia	373.72 (280.52-477.22)	506.72 (349.75-670.83)	0.36	18.4 (13.73-23.63)	15.14 (10.49-20.07)	-0.78 (-0.91--0.64)
Madagascar	579.3	779.16	0.35	9.63	4.95	-2.44

	(197.26-896.01)	(311.86-1378.49)		(3.58-14.95)	(1.87-8.52)	(-2.99--1.88)
Malawi	230.91 (103.68-358.85)	812.87 (474.8-1214.43)	2.52	4.91 (2.25-7.6)	8.33 (4.88-12.29)	1.79 (1.72-1.87)
Malaysia	818.33 (487.44-1220.47)	1225.87 (660.3-1927.49)	0.5	7.53 (4.46-11.22)	3.91 (2.09-6.13)	-2.8 (-3.33--2.26)
Maldives	0.75 (0-2.69)	5.59 (1.84-11.97)	6.45	0.69 (0-2.49)	1.18 (0.38-2.62)	-1.85 (-3.92-0.26)
Mali	91.16 (47.88-138.44)	240.77 (135.68-378.97)	1.64	2.02 (1.05-3.08)	2.3 (1.27-3.56)	0.5 (0.45-0.55)
Malta	53.37 (39.92-68.18)	78.48 (58.56-100.58)	0.47	12.57 (9.41-16.03)	10.64 (7.97-13.59)	-0.48 (-0.63--0.32)
Marshall Islands	0.81 (0.34-1.43)	2.85 (1.3-5.2)	2.52	3.51 (1.45-6.16)	5.9 (2.74-10.69)	1.8 (1.71-1.89)
Mauritius	123.4 (82.28-158.31)	253.28 (176.06-328.66)	1.05	15.18 (10.14-19.51)	13.66 (9.44-17.76)	-0.23 (-0.62-0.17)
Mexico	2822.88 (2170.03-3453.3)	6704.25 (4961.32-8495.81)	1.37	5.79 (4.4-7.16)	5.02 (3.7-6.39)	-0.71 (-0.92--0.5)
Micronesia (Federated States of)	4.69 (2.25-7.5)	5.36 (2.57-8.93)	0.14	8.07 (3.88-13.06)	5.77 (2.77-9.56)	-1.31 (-1.41--1.21)
Moldova	1905.23 (1448.55-2297.39)	1838.88 (1383.4-2302.48)	-0.03	40.86 (31.1-49.52)	32.46 (24.59-40.39)	-1.14 (-1.48--0.79)
Monaco	3.03 (0.07-5.69)	4.35 (0.16-8.5)	0.44	5.86 (0.15-10.91)	6.13 (0.27-12.01)	0.17 (0.09-0.26)
Mongolia	122.43 (66.38-190.81)	377.32 (242.17-543.85)	2.08	10.28 (5.47-16.14)	12.74 (8.05-18.41)	1.43 (1.16-1.7)
Montenegro	155.14 (111.55-204.15)	219.58 (154.05-284.99)	0.42	23.35 (16.71-30.61)	23.79 (16.67-31.05)	0.01 (-0.12-0.15)
Morocco	52.49 (32.22-78.16)	83.97 (46.1-140.93)	0.6	0.29 (0.18-0.44)	0.21 (0.12-0.35)	-0.87 (-0.98--0.76)

Mozambique	64.33 (4.23-152.59)	706.37 (306.7-1129.87)	9.98	0.9 (0.06-2.13)	5.02 (2.22-8.07)	5.75 (5.59-5.91)
Myanmar	252.77 (77.02-557.64)	3680.77 (2268.06-5592.09)	13.56	0.91 (0.27-2.05)	6.72 (4.16-10.12)	7.96 (7.46-8.46)
Namibia	209.4 (97.36-328.57)	854.55 (535-1235.21)	3.08	27.11 (12.56-42.76)	50.19 (31.39-71.83)	2.07 (1.8-2.34)
Nauru	0.54 (0.21-1.04)	0.94 (0.46-1.8)	0.74	8.71 (3.38-16.6)	12.14 (6.01-22.82)	1.33 (1.19-1.46)
Nepal	122.21 (3.87-454.78)	4826.14 (1902.09-8496.92)	38.49	0.98 (0.03-3.72)	18.4 (7.21-32.28)	9.44 (8.11-10.78)
Netherlands	3119.68 (2458.32-3739.82)	3534.05 (2779.6-4283.49)	0.13	16.9 (13.33-20.18)	11.27 (8.9-13.63)	-1.22 (-1.44--0.99)
New Zealand	697.21 (491.39-882.01)	970.22 (779.59-1182.17)	0.39	18.49 (13.22-23.24)	12.68 (10.19-15.36)	-0.97 (-1.27--0.67)
Nicaragua	47.36 (33.25-63.17)	147.54 (102.94-199.31)	2.12	2.61 (1.8-3.51)	2.71 (1.89-3.69)	0.38 (0.16-0.6)
Niger	2.62 (0.08-8.18)	25.84 (5.13-64.42)	8.86	0.07 (0-0.22)	0.25 (0.05-0.62)	5.48 (4.46-6.5)
Nigeria	1435.39 (845.94-2032.88)	4203.81 (2664.98-6138.67)	1.93	2.88 (1.71-4.06)	3.75 (2.44-5.37)	0.86 (0.77-0.96)
Niue	0.13 (0.03-0.23)	0.21 (0.1-0.31)	0.62	6.55 (1.33-11)	9.98 (4.76-15.03)	0.81 (0.51-1.11)
Northern Mariana Islands	2.93 (0.06-6.74)	16.91 (1.78-29.86)	4.77	9.02 (0.13-21.35)	27.62 (2.96-49.14)	4.2 (3.82-4.57)
Norway	617.95 (435.87-811.23)	699.65 (532.86-888.45)	0.13	10.99 (8-14.27)	7.89 (6.09-9.94)	-1.05 (-1.34--0.77)
Oman	4.72 (2.09-8.65)	17.16 (8.45-29.34)	2.64	0.45 (0.18-0.84)	0.46 (0.23-0.79)	-0.3 (-1.38-0.78)
Pakistan	3646.57 (750.85-6955.18	20817.93 (9213.33-34525.7	4.71	5.46 (1.09-10.4	12.4 (5.32-20.6	2.92 (2.65-3.2)

	)	8)		2)	2)	
Palau	5.38 (0.71-11.53)	15.95 (4.04-30.62)	1.96	44.31 (5.74-96.13)	62.82 (16.37-119.17)	1.15 (1.05-1.25)
Palestine	6.4 (3.39-9.82)	17.63 (10.95-26.61)	1.75	0.61 (0.3-0.95)	0.52 (0.32-0.78)	-0.78 (-0.99--0.58)
Panama	131.65 (99.58-164.33)	275.87 (185.48-367.68)	1.1	8.27 (6.19-10.41)	6.25 (4.2-8.33)	-0.81 (-0.99--0.62)
Papua New Guinea	62.77 (27.29-104.44)	185.78 (90.12-300.65)	1.96	2.47 (1.1-4.12)	2.48 (1.16-4.02)	0.6 (-0.01-1.22)
Paraguay	331.85 (233.18-440.29)	1007.22 (662.03-1465.9)	2.04	13.83 (9.72-18.35)	16.1 (10.54-23.48)	0.29 (0.15-0.43)
Peru	744.16 (437.71-1059.43)	1681.22 (1066.77-2491.01)	1.26	5.45 (3.28-7.84)	4.79 (3.04-7.11)	-0.25 (-0.64-0.15)
Philippines	5022.08 (3557.4-6555.52)	12182.12 (8630.7-16222.82)	1.43	13.63 (9.49-17.96)	12.75 (9.08-16.99)	-0.36 (-0.44--0.28)
Poland	11613.45 (9073.88-14046.95)	21242.94 (16998.75-25425.29)	0.83	27.42 (21.45-33.15)	34.66 (27.82-41.32)	0.78 (0.69-0.88)
Portugal	4398.3 (3516.64-5186.89)	4877.37 (3909.91-5917.66)	0.11	34.42 (27.67-40.4)	26.12 (21-31.54)	-0.92 (-1.08--0.77)
Puerto Rico	683.57 (499.14-859.31)	458.48 (316.06-619.39)	-0.33	19.16 (14.05-24.07)	8.43 (5.86-11.23)	-2.3 (-2.53--2.07)
Qatar	1.49 (0.84-2.32)	11.88 (6.15-18.82)	6.97	0.56 (0.31-0.86)	0.48 (0.22-0.79)	0.17 (-0.48-0.83)
Romania	8034.79 (6152.36-9863.95)	14124.47 (11009.79-17729.2)	0.76	28.78 (22.17-35.19)	45.89 (35.59-57.55)	1.58 (1.24-1.92)
Russian Federation	56621.94 (43670.46-67665.77)	62124.32 (47336.05-76649.21)	0.1	31.21 (24.27-37.2)	28.77 (22.65-35.43)	-1 (-1.32--0.67)
Rwanda	1124.56 (624.01-1594.36)	1633.9 (899.25-2553.05)	0.45	32.49 (18.66-46.23)	20.5 (11.24-32.05)	-2.32 (-2.6--2.04)
Saint Kitts and	4.26 (0.05-5.86)	5.43 (0.04-12.7)	0.27	13.07	6.82	-1.93

Nevis				(0.16-18.01)	(0.06-16.07)	(-2.35--1.51)
Saint Lucia	26.6 (20.86-32.19)	52.4 (37.42-68.44)	0.97	30.03 (23.49-36.62)	21.48 (15.39-28.02)	-1.03 (-1.23--0.84)
Saint Vincent and the Grenadines	16.96 (11.81-22.23)	46.65 (34.92-60.31)	1.75	23.82 (16.54-31.34)	32.27 (24.13-41.62)	1.22 (0.92-1.52)
Samoa	6.19 (3.02-9.41)	7.81 (3.83-13.1)	0.26	6.34 (3.07-9.66)	4.72 (2.32-7.88)	-1.17 (-1.5--0.84)
San Marino	5.15 (0.02-8.02)	5.25 (0.03-9.21)	0.02	16.47 (0.07-25.37)	8.74 (0.06-15.41)	-1.14 (-1.46--0.82)
Sao Tome and Principe	0.17 (0.09-0.24)	0.5 (0.31-0.76)	1.94	0.25 (0.14-0.36)	0.38 (0.24-0.58)	0.72 (0.38-1.07)
Saudi Arabia	35.17 (9.54-63.95)	72.54 (10.25-153.22)	1.06	0.43 (0.11-0.81)	0.2 (0.02-0.43)	-1.1 (-1.73--0.46)
Senegal	25.28 (5.8-52.9)	51.63 (12.88-115.18)	1.04	0.68 (0.16-1.4)	0.55 (0.14-1.22)	-1.21 (-1.6--0.82)
Serbia	3073.39 (2061.58-4124.34)	3280.87 (2390.75-4288.13)	0.07	25.35 (17.06-34.25)	23.09 (16.97-30.02)	-0.36 (-0.51--0.21)
Seychelles	19.29 (13.2-25.46)	51.41 (35.8-66.84)	1.67	34.58 (23.81-45.74)	39.74 (27.86-51.94)	0.44 (-0.03-0.91)
Sierra Leone	87.53 (49.39-126.08)	203.43 (121.53-314.86)	1.32	3.98 (2.25-5.71)	4.44 (2.66-6.78)	0.25 (0.16-0.34)
Singapore	88.6 (57.99-121.16)	171.62 (115.55-229.55)	0.94	3.29 (2.19-4.5)	2.01 (1.36-2.69)	-2.16 (-2.69--1.64)
Slovakia	4498.92 (3508.05-5639.36)	4164.13 (2821.85-5679.18)	-0.07	79.16 (61.82-99.36)	48.75 (33.06-66.64)	-1.69 (-1.77--1.61)
Slovenia	820.36 (613.41-1014.2)	442.91 (226.77-687.44)	-0.46	33.62 (25.24-41.54)	12.01 (6.18-18.79)	-3.69 (-3.89--3.49)
Solomon Islands	1.42 (0.34-2.88)	11.64 (4.59-21.5)	7.2	0.74 (0.16-1.5)	2.31 (0.91-4.32)	5.26 (4.35-6.18)

South Africa	6963.81 (4355.28-9273.43)	11837.87 (8364.59-14836.32)	0.7	29.64 (18.36-39.93)	22.57 (15.74-28.3)	-1.36 (-1.58--1.13)
South Korea	3496.81 (2724.29-4358.48)	6020.94 (4278.26-7845.59)	0.72	9.61 (7.45-12.16)	6.87 (4.89-9.03)	-1.55 (-1.77--1.32)
South Sudan	24.45 (0.73-63.69)	25.62 (2.97-70.36)	0.05	0.82 (0.03-2.15)	0.47 (0.06-1.3)	-1.71 (-1.99--1.43)
Spain	16730.35 (13300.11-20156.25)	13384.88 (10662.27-16274.78)	-0.2	34.73 (28.05-41.75)	16.53 (13.2-19.91)	-2.84 (-3--2.67)
Sri Lanka	1644.9 (1089.01-2257.87)	5832.31 (3166.7-9157.84)	2.55	13.55 (8.91-18.65)	21.18 (11.6-33.17)	2.08 (1.67-2.49)
Sudan	159.13 (94.02-264.03)	0.09 (0-0.62)	-1	1.45 (0.85-2.39)	0 (0-0)	-20.63 (-25.18--15.81)
Suriname	25.27 (17.73-32.29)	45.5 (28.91-69.47)	0.8	8.86 (6.24-11.42)	6.75 (4.3-10.31)	-0.45 (-0.64--0.27)
Swaziland	67.54 (43.28-101.09)	139.9 (75.92-235.84)	1.07	19.19 (12.39-28.13)	19.8 (10.79-32.97)	0 (-0.33-0.33)
Sweden	1521.39 (1174.12-1841.08)	1735.16 (1303.8-2170.32)	0.14	12.25 (9.64-14.72)	9.79 (7.41-12.18)	-0.07 (-0.37-0.24)
Switzerland	1767.32 (1428.83-2131.1)	1796.92 (1367.51-2235.17)	0.02	19.4 (15.81-23.48)	11.5 (8.79-14.22)	-1.78 (-2.09--1.46)
Syria	40.92 (25.16-62.74)	43.87 (20.21-74.66)	0.07	0.64 (0.37-0.99)	0.29 (0.13-0.5)	-2.76 (-3.12--2.39)
Taiwan (Province of China)	5165.56 (4121.53-6142.67)	20023.72 (15697.86-25292.35)	2.88	28.18 (22.48-33.53)	52.67 (41.29-66.48)	1.5 (0.92-2.09)
Tajikistan	83.12 (49.81-129.47)	123.34 (71.86-193.95)	0.48	2.62 (1.55-4.05)	1.5 (0.88-2.37)	-2.08 (-2.38--1.78)
Tanzania	2480.46 (1342.56-3706.2)	5176.78 (3054.11-7688.49)	1.09	19.75 (11-29.14)	16.94 (10.19-25.13)	-0.7 (-0.77--0.63)
Thailand	8630.9 (5964.94-11567.	19478.85 (13606.86-27351.	1.26	20.18 (13.98-27.	19 (13.36-26.	-0.57 (-0.7--0.44)

	57)	86)		04)	41)	
The Bahamas	50.15 (37.88-62.45)	78.44 (44.8-115.49)	0.56	28.59 (21.33-35.96)	17.24 (9.79-25.24)	-1.54 (-1.8--1.28)
The Gambia	7.84 (2.68-14.38)	53.3 (28.96-83.79)	5.8	1.81 (0.63-3.32)	4.44 (2.43-6.95)	2.67 (2.12-3.22)
Timor-Leste	12.94 (5.88-22.38)	61.99 (37.56-92.19)	3.79	3.39 (1.51-5.95)	6.72 (4.04-9.99)	1.95 (1.67-2.24)
Togo	49.09 (24.54-75.25)	206.66 (111.75-333.12)	3.21	3.19 (1.64-4.84)	4.05 (2.19-6.45)	0.99 (0.74-1.23)
Tokelau	0.05 (0.02-0.1)	0.11 (0.05-0.18)	1.2	4.2 (1.55-7.61)	7.44 (3.55-12.49)	2.03 (1.95-2.11)
Tonga	1.2 (0.45-2.1)	1.69 (0.5-3.43)	0.41	1.9 (0.71-3.33)	1.95 (0.57-3.97)	-0.12 (-0.94-0.7)
Trinidad and Tobago	99.46 (72.52-126.36)	178.61 (121.52-258.02)	0.8	11.29 (8.17-14.37)	9.37 (6.36-13.48)	-0.25 (-0.43--0.07)
Tunisia	123.67 (76.83-181.53)	346.18 (209.92-548.49)	1.8	2.13 (1.3-3.12)	2.47 (1.5-3.89)	0.25 (0.15-0.35)
Turkey	848.9 (525.66-1255.96)	1419.46 (859.13-2058.1)	0.67	2.02 (1.24-2.99)	1.45 (0.88-2.11)	-1.68 (-1.92--1.44)
Turkmenistan	235.23 (130.1-360.82)	702.1 (440.3-971.22)	1.98	10.5 (5.67-16.39)	14.62 (9.09-20.38)	0.82 (0.55-1.1)
Tuvalu	0.21 (0.08-0.41)	0.55 (0.25-0.95)	1.62	2.76 (0.99-5.29)	4.8 (2.18-8.3)	1.76 (1.31-2.2)
Uganda	1632.14 (813.15-2321.42)	5417.67 (3309.92-7786.93)	2.32	21.21 (10.94-30.23)	27.49 (16.97-39.14)	0.38 (0.15-0.61)
Ukraine	17319.46 (12115.99-22956.02)	21623.27 (12650.85-32063.6)	0.25	24.76 (17.47-32.98)	31.93 (18.77-46.94)	0.9 (0.42-1.38)
United Arab Emirates	58.61 (35.19-96.07)	254.23 (153.57-385.35)	3.34	5.68 (3.01-10.03)	2.76 (1.6-4.35)	-1.91 (-2.14--1.68)
United Kingdom	11610.87 (9092.93-13999.	17705.12 (14135.62-21231.	0.52	15.01 (11.96-17.	16.36 (13.17-19.	0.32 (0.24-0.41)

	08)	4)		89)	5)	
United States	43879.52 (31186.83-56714.56)	58096.73 (44807.06-70597.53)	0.32	15.33 (11-19.65)	11 (8.54-13.32)	-0.89 (-1.09--0.68)
Uruguay	923.06 (723.76-1143.25)	909.26 (709.95-1150.87)	-0.01	25.11 (19.76-31.1)	19.15 (14.98-24.16)	-0.81 (-0.93--0.69)
Uzbekistan	723.22 (436.9-1024.32)	2548.7 (1668.93-3539.79)	2.52	5.52 (3.31-7.83)	7.85 (5.04-10.89)	0.79 (0.52-1.06)
Vanuatu	2.77 (1.27-4.73)	11.44 (5.9-19.15)	3.13	3.21 (1.48-5.5)	4.92 (2.56-8.11)	1.9 (0.71-3.1)
Venezuela	1081.6 (852.62-1315.6)	2230.82 (1463.84-3258.02)	1.06	9.82 (7.65-12.06)	7.19 (4.75-10.46)	-1.18 (-1.38--0.98)
Viet Nam	430.4 (59.3-1040.82)	30232.83 (20615.91-41992.57)	69.24	0.97 (0.13-2.38)	27.19 (18.69-37.35)	10.96 (9.47-12.47)
Virgin Islands, U.S.	10.01 (0.03-20.14)	11.94 (0.59-21.65)	0.19	10.3 (0.03-20.8)	8.37 (0.45-14.72)	-0.46 (-0.65--0.28)
Yemen	59.31 (30.17-101.16)	50.45 (27.93-86.23)	-0.15	0.95 (0.48-1.62)	0.25 (0.14-0.44)	-4.87 (-5.16--4.58)
Zambia	573.91 (263.81-837.63)	3193.27 (1110.85-5624.51)	4.56	16.21 (7.86-23.47)	32.8 (11.93-57.66)	2.97 (2.32-3.63)
Zimbabwe	482.34 (266.61-674.69)	1234.21 (732.6-1812.44)	1.56	10.07 (5.59-14.23)	13.35 (7.85-19.43)	1.03 (0.84-1.22)

Supplementary table 1. The DALYs of lip and oral cavity cancer attributable to high alcohol consumption cases and rates in 1990 and 2021 across 204 countries, and the trends from 1990 to 2021.

Location	1990_Death cases (95% UI)	2021_Death cases (95% UI)	Percentage change	1990_ASMR_per 100000(95% UI)	2021_ASMR_per 100 000(95% UI)	EAPC (95% CI)
Albania	3.81 (1.94-6.07)	13.43 (8.24-21.28)	2.52	0.17 (0.09-0.28)	0.32 (0.2-0.51)	2.21 (1.55-2.89)
Algeria	2.45 (1.26-3.78)	9.19 (5.2-14.89)	2.75	0.02 (0.01-0.03)	0.02 (0.01-0.04)	1.17 (1.02-1.31)
American Samoa	0 (0-0)	0 (0-0.01)	NA	0 (0-0.02)	0.01 (0-0.02)	1.42 (0.36-2.5)
Andorra	0.32 (0.2-0.48)	0.5 (0.3-0.74)	0.56	0.55 (0.34-0.82)	0.32 (0.2-0.48)	-1.54 (-1.71--1.38)
Angola	9.46 (3.8-16.25)	57.13 (33.7-83.14)	5.04	0.21 (0.08-0.35)	0.43 (0.26-0.62)	3.03 (2.55-3.51)
Antigua and Barbuda	0.11 (0.05-0.18)	0.43 (0.31-0.55)	2.91	0.22 (0.1-0.35)	0.39 (0.28-0.5)	1.81 (1.48-2.15)
Argentina	234.06 (184.96-285.19)	219.47 (161.89-276.22)	-0.06	0.72 (0.57-0.88)	0.4 (0.3-0.5)	-1.4 (-1.66--1.14)
Armenia	4.66 (2.81-6.81)	9.58 (6.05-13.28)	1.06	0.15 (0.09-0.23)	0.22 (0.14-0.31)	1.62 (1.23-2.02)
Australia	126.59 (83.75-164.44)	221.26 (164.25-277.24)	0.75	0.66 (0.44-0.85)	0.5 (0.38-0.62)	-0.79 (-1--0.57)
Austria	101.86 (79.31-122.83)	108.24 (84.3-132.5)	0.06	0.96 (0.76-1.14)	0.64 (0.5-0.78)	-1.27 (-1.38--1.16)
Azerbaijan	8.14 (4.66-13.26)	15.85 (8.09-26.15)	0.95	0.15 (0.09-0.24)	0.14 (0.07-0.23)	0.38 (0.09-0.67)
Bahrain	0.21 (0.14-0.31)	0.47 (0.24-0.71)	1.24	0.1 (0.06-0.14)	0.04 (0.02-0.06)	-3.18 (-3.47--2.89)
Bangladesh	9.17 (0-31.97)	117.59 (4.19-270.43)	11.82	0.02 (0-0.06)	0.08 (0-0.18)	4.95 (4.37-5.53)
Barbados	1.41 (1.02-1.79)	2.56 (1.82-3.61)	0.82	0.52 (0.38-0.66)	0.5 (0.36-0.71)	0.22 (0.05-0.38)

Belarus	207.82 (147.33-262.7)	191.94 (129.86-262.92)	-0.08	1.59 (1.14-2.02)	1.26 (0.86-1.72)	-0.96 (-1.16--0.77)
Belgium	121.77 (92.78-147.29)	141.93 (110.86-174.44)	0.17	0.86 (0.67-1.04)	0.67 (0.53-0.82)	-0.89 (-1.12--0.65)
Belize	0.2 (0.14-0.25)	0.67 (0.47-0.87)	2.35	0.21 (0.15-0.27)	0.21 (0.15-0.27)	0.46 (0.05-0.88)
Benin	1.16 (0.5-1.95)	6.05 (2.99-10.02)	4.22	0.06 (0.02-0.09)	0.1 (0.05-0.17)	2.01 (1.86-2.16)
Bermuda	0.82 (0.62-1.01)	0.83 (0.58-1.1)	0.01	1.31 (0.98-1.61)	0.63 (0.45-0.83)	-1.91 (-2.26--1.56)
Bhutan	2.27 (1.01-3.62)	0.72 (0.17-1.62)	-0.68	0.79 (0.36-1.27)	0.11 (0.02-0.25)	-8.12 (-8.77--7.47)
Bolivia	7.42 (3.91-11.7)	19.98 (12.75-31.02)	1.69	0.22 (0.12-0.35)	0.21 (0.13-0.33)	0.18 (-0.05-0.42)
Bosnia and Herzegovina	17 (11.03-23.05)	31.86 (22.59-42.91)	0.87	0.37 (0.24-0.51)	0.54 (0.38-0.72)	1.41 (1.29-1.54)
Botswana	3.59 (1.96-5.6)	8.72 (4.62-13.49)	1.43	0.57 (0.31-0.89)	0.51 (0.27-0.77)	-0.85 (-1.16--0.54)
Brazil	545.6 (431.82-676.3)	1373.9 (1061.93-1718.61)	1.52	0.57 (0.45-0.71)	0.53 (0.41-0.67)	-0.27 (-0.55-0.02)
Brunei	0.2 (0.13-0.29)	0.13 (0.01-0.27)	-0.35	0.16 (0.1-0.24)	0.03 (0-0.07)	-1.52 (-3.45-0.46)
Bulgaria	75.11 (56.37-93.96)	119.77 (88.39-156.78)	0.59	0.64 (0.48-0.81)	0.98 (0.72-1.28)	0.7 (0.39-1.02)
Burkina Faso	6.83 (3.98-9.54)	21.2 (13.47-30.22)	2.1	0.15 (0.09-0.21)	0.22 (0.14-0.31)	0.94 (0.73-1.15)
Burundi	23.74 (13.96-33.43)	30.66 (17.7-46.6)	0.29	0.96 (0.58-1.35)	0.56 (0.32-0.84)	-2.24 (-2.42--2.06)
Cambodia	5.3 (2.19-9.99)	73.61 (48.83-109.34)	12.89	0.11 (0.04-0.2)	0.57 (0.38-0.83)	5.05 (4.7-5.41)

Cameroon	8.23 (4.39-12.17)	42.17 (26.86-61.48)	4.12	0.17 (0.09-0.26)	0.3 (0.19-0.45)	2.21 (2.07-2.34)
Canada	189.33 (124.99-258.09)	282.39 (204.94-373.16)	0.49	0.6 (0.4-0.81)	0.4 (0.3-0.53)	-0.83 (-1.01--0.65)
Cape Verde	0.05 (0.03-0.07)	3.11 (1.92-4.81)	61.2	0.02 (0.01-0.03)	0.64 (0.4-0.99)	8.9 (6.31-11.56)
Central African Republic	3.74 (1.59-7.32)	4.33 (1.65-8.73)	0.16	0.3 (0.14-0.57)	0.17 (0.06-0.33)	-1.7 (-1.79--1.61)
Chad	0.78 (0.07-2.12)	8.77 (2.29-16.98)	10.24	0.03 (0-0.07)	0.13 (0.03-0.26)	6.45 (5.92-6.99)
Chile	39.03 (31.58-47.97)	61.51 (47.78-77.6)	0.58	0.38 (0.31-0.47)	0.24 (0.19-0.3)	-1.16 (-1.29--1.03)
China	2233.14 (1609.24-2866.6)	6360.63 (4458.62-8712.79)	1.85	0.26 (0.18-0.33)	0.3 (0.21-0.41)	0.84 (0.47-1.21)
Colombia	50.33 (38.71-64.3)	72.31 (47.12-102.82)	0.44	0.27 (0.2-0.35)	0.13 (0.09-0.19)	-2.97 (-3.32--2.62)
Comoros	0.06 (0-0.15)	0.35 (0.1-0.71)	4.83	0.03 (0-0.07)	0.07 (0.02-0.14)	3.77 (3.51-4.03)
Congo	3.23 (1.11-5.95)	14.33 (7.82-21.2)	3.44	0.29 (0.11-0.53)	0.5 (0.28-0.73)	2.64 (1.93-3.35)
Cook Islands	0.01 (0-0.02)	0.1 (0.07-0.14)	9	0.05 (0-0.15)	0.41 (0.27-0.56)	8.57 (7.5-9.65)
Costa Rica	6.5 (4.98-8.14)	11.56 (8.2-15.12)	0.78	0.37 (0.28-0.46)	0.21 (0.15-0.27)	-2.34 (-2.66--2.03)
Cote d'Ivoire	13.16 (6.42-20.29)	53.6 (28.53-83.19)	3.07	0.29 (0.14-0.46)	0.42 (0.24-0.64)	0.9 (0.74-1.06)
Croatia	96.4 (72.19-118.51)	72.6 (55.18-90.97)	-0.25	1.53 (1.14-1.89)	0.89 (0.68-1.12)	-1.78 (-1.9--1.66)
Cuba	51.33 (35.36-67.95)	122.94 (85.71-159.47)	1.4	0.5 (0.34-0.6	0.63 (0.44-0.8	1.21 (1.03-1.4)

				7)	2)	
Cyprus	3.45 (2.41-4.56)	5.95 (4.19-7.95)	0.72	0.48 (0.33-0.65)	0.3 (0.21-0.4)	-1.39 (-1.5--1.27)
Czech Republic	159.52 (122.33-193.59)	193.42 (151.54-239.29)	0.21	1.21 (0.94-1.46)	0.99 (0.78-1.23)	-0.63 (-0.73--0.53)
Democratic People's Republic of Korea	39.82 (24.71-57.98)	74.1 (45.19-111.8)	0.86	0.22 (0.14-0.32)	0.22 (0.13-0.32)	0.11 (-0.05-0.26)
Democratic Republic of the Congo	32.61 (11.69-54.85)	76.26 (30.57-129.67)	1.34	0.2 (0.08-0.33)	0.19 (0.08-0.32)	0.11 (-1.61-1.86)
Denmark	41 (31.19-49.42)	60.77 (46.91-74.13)	0.48	0.55 (0.43-0.67)	0.54 (0.42-0.66)	-0.86 (-1.46--0.25)
Djibouti	0.27 (0.07-0.55)	0.15 (0.03-0.39)	-0.44	0.16 (0.05-0.31)	0.02 (0-0.05)	-8.25 (-9--7.49)
Dominica	0.4 (0.29-0.53)	0.55 (0.36-0.77)	0.38	0.68 (0.49-0.9)	0.64 (0.42-0.89)	-0.09 (-0.17-0)
Dominican Republic	14.52 (9.87-20.54)	42.72 (26.03-67.14)	1.94	0.37 (0.25-0.53)	0.42 (0.26-0.66)	0.39 (0.18-0.6)
Ecuador	5.22 (3.22-7.08)	17.23 (11.61-24.39)	2.3	0.09 (0.05-0.12)	0.1 (0.07-0.15)	1.45 (1-1.89)
Egypt	0.7 (0.38-1.08)	3.75 (2.17-5.55)	4.36	0 (0-0)	0.01 (0-0.01)	4.18 (3.44-4.92)
El Salvador	4.89 (3.42-6.37)	9.15 (6.42-12.97)	0.87	0.16 (0.11-0.21)	0.15 (0.1-0.21)	-0.75 (-1.03--0.48)
Equatorial Guinea	0.44 (0.14-0.88)	2.63 (1.46-3.96)	4.98	0.21 (0.07-0.41)	0.48 (0.28-0.71)	2.91 (2.61-3.21)
Eritrea	2.69 (0.65-5.21)	4.23 (1.37-7.87)	0.57	0.18 (0.04-0.34)	0.12 (0.04-0.22)	-2.92 (-3.43--2.4)
Estonia	13.91 (9.5-18.74)	18.57 (12.03-25.23)	0.34	0.69 (0.48-0.93)	0.81 (0.55-1.07)	0.03 (-0.28-0.35)
Ethiopia	30.32 (9.25-65.75)	131.5 (73.16-207.52)	3.34	0.14 (0.04-0.3)	0.29 (0.16-0.4	2.6 (1.85-3.36)

					7)	
Fiji	0.6 (0.3-0.92)	1.5 (0.8-2.23)	1.5	0.14 (0.07-0.21)	0.18 (0.1-0.26)	1.15 (0.74-1.56)
Finland	29.47 (22.23-36.14)	39.03 (29.31-48.92)	0.32	0.44 (0.34-0.54)	0.35 (0.27-0.44)	-0.46 (-0.6--0.32)
France	1381.84 (1080.35-1671.46)	925.99 (720.28-1132.25)	-0.33	1.84 (1.45-2.23)	0.73 (0.58-0.88)	-3.05 (-3.24--2.85)
Gabon	3.85 (2.22-5.78)	6.66 (3.89-9.31)	0.73	0.68 (0.4-1)	0.61 (0.37-0.86)	-0.49 (-0.58--0.41)
Georgia	23 (13.48-33.73)	36.97 (24.05-49.49)	0.61	0.36 (0.21-0.52)	0.65 (0.43-0.86)	3.3 (2.52-4.08)
Germany	1297.08 (1029.87-1540.72)	1222.04 (966.52-1477.81)	-0.06	1.12 (0.9-1.33)	0.69 (0.55-0.82)	-1.51 (-1.67--1.34)
Ghana	3.22 (1.61-5.1)	6.69 (3.33-10.74)	1.08	0.05 (0.02-0.07)	0.04 (0.02-0.06)	-1.7 (-2.17--1.23)
Greece	58.4 (43.93-70.95)	88.18 (66.7-108.65)	0.51	0.4 (0.3-0.48)	0.42 (0.32-0.51)	0.02 (-0.13-0.18)
Greenland	0.46 (0.27-0.67)	0.65 (0.4-0.9)	0.41	1.25 (0.69-1.91)	0.87 (0.54-1.21)	-0.94 (-1.08--0.81)
Grenada	0.48 (0.34-0.63)	0.71 (0.49-0.93)	0.48	0.72 (0.52-0.93)	0.59 (0.4-0.77)	-0.61 (-0.96--0.25)
Guam	0.05 (0-0.14)	0.55 (0.12-0.96)	10	0.06 (0-0.15)	0.28 (0.06-0.49)	7.07 (6.48-7.66)
Guatemala	5.95 (4.39-7.74)	10.4 (7.09-14.9)	0.75	0.17 (0.12-0.22)	0.09 (0.06-0.13)	-2.42 (-2.65--2.19)
Guinea	3.86 (1.35-7.2)	14.85 (6.22-25.69)	2.85	0.11 (0.04-0.21)	0.24 (0.1-0.42)	1.86 (1.11-2.62)
Guinea-Bissau	0.61 (0.29-1.03)	1.61 (0.93-2.58)	1.64	0.14 (0.07-0.24)	0.19 (0.11-0.3)	0.84 (0.58-1.1)
Guyana	1.78	2.16 (1.46-3.07)	0.21	0.44	0.31	-0.56

	(1.31-2.28)			(0.32-0.57)	(0.21-0.44)	(-0.75--0.38)
Haiti	19.98 (12.46-33.96)	40.04 (24.58-61.46)	1	0.59 (0.37-0.99)	0.53 (0.33-0.83)	-0.19 (-0.23--0.15)
Honduras	2.33 (1.54-3.15)	8.73 (5.57-13.37)	2.75	0.11 (0.07-0.15)	0.14 (0.09-0.21)	0.64 (0.51-0.76)
Hungary	304.9 (231.66-375.44)	275.63 (208.73-359.05)	-0.1	2.21 (1.69-2.72)	1.61 (1.23-2.09)	-1.47 (-1.79--1.15)
Iceland	0.86 (0.57-1.15)	2.24 (1.65-2.83)	1.6	0.32 (0.21-0.43)	0.4 (0.3-0.51)	0.92 (0.81-1.03)
India	2653.02 (1169.88-3739.1)	10438.68 (6901.66-14271.95)	2.93	0.5 (0.23-0.7)	0.82 (0.53-1.13)	1.88 (1.62-2.13)
Indonesia	27.45 (8.25-49.56)	64.73 (17.73-129.59)	1.36	0.02 (0.01-0.04)	0.02 (0.01-0.05)	-0.64 (-0.97--0.31)
Iran	0 (0-0)	8.89 (5.57-12.93)	Inf	0 (0-0)	0.01 (0.01-0.02)	41.04 (33.18-49.36)
Iraq	1.06 (0.65-1.55)	2.12 (1.26-3.28)	1	0.01 (0.01-0.02)	0.01 (0-0.01)	-1.73 (-1.93--1.54)
Ireland	32.37 (24.29-40.35)	34.53 (27.04-42.5)	0.07	0.82 (0.63-1.02)	0.45 (0.35-0.56)	-2.01 (-2.19--1.82)
Israel	2.76 (1.28-4.44)	11.62 (7.43-16.45)	3.21	0.06 (0.03-0.1)	0.1 (0.06-0.14)	2.05 (1.77-2.34)
Italy	889.54 (685.17-1056.26)	775.51 (589.45-943.27)	-0.13	1.05 (0.81-1.24)	0.55 (0.43-0.67)	-2.17 (-2.38--1.96)
Jamaica	3.11 (2.05-4.28)	5.84 (3.73-8.75)	0.88	0.18 (0.12-0.25)	0.19 (0.12-0.28)	0.36 (-0.11-0.83)
Japan	515.54 (404.41-620.37)	1151.13 (822.61-1452.26)	1.23	0.31 (0.24-0.37)	0.33 (0.24-0.4)	-0.33 (-0.74-0.08)
Jordan	0.22 (0.13-0.34)	1.1 (0.6-1.73)	4	0.01 (0.01-0.02)	0.01 (0.01-0.02)	-0.17 (-0.65-0.31)

Kazakhstan	106.71 (72.52-137.53)	89.06 (59.58-118.97)	-0.17	0.78 (0.53-1.01)	0.46 (0.31-0.62)	-2.35 (-2.57--2.12)
Kenya	51.48 (25.21-75.78)	172.02 (108.65-237.6)	2.34	0.59 (0.29-0.87)	0.67 (0.43-0.91)	0.64 (0.47-0.81)
Kiribati	0.15 (0.03-0.3)	0.17 (0.02-0.43)	0.13	0.34 (0.06-0.7)	0.19 (0.02-0.49)	-2.45 (-2.85--2.05)
Kyrgyzstan	26.21 (17.87-34.25)	15.15 (10.56-20.73)	-0.42	0.83 (0.57-1.09)	0.27 (0.19-0.38)	-1.98 (-2.74--1.21)
Lao People's Democratic Republic	7.23 (3.51-12.39)	23.66 (14.83-33.22)	2.27	0.32 (0.15-0.54)	0.48 (0.3-0.68)	1.62 (1.18-2.05)
Latvia	31.85 (22.51-41.48)	41.79 (29.3-56.02)	0.31	0.91 (0.65-1.18)	1.24 (0.89-1.63)	0.79 (0.59-1)
Lebanon	2.96 (1.58-5.18)	4.21 (2.37-6.65)	0.42	0.13 (0.07-0.22)	0.07 (0.04-0.11)	-1.94 (-2.06--1.82)
Lesotho	3.78 (2.03-5.89)	8.75 (4.3-15.42)	1.31	0.42 (0.22-0.66)	0.73 (0.36-1.27)	2.03 (1.83-2.24)
Liberia	1.84 (1.05-2.78)	4.35 (2.52-6.8)	1.36	0.16 (0.09-0.23)	0.17 (0.1-0.26)	-0.03 (-0.15-0.09)
Libya	0 (0-0.01)	1.09 (0.61-1.78)	Inf	0 (0-0)	0.02 (0.01-0.03)	11.94 (7.71-16.34)
Lithuania	36.44 (24.99-46.74)	60.5 (42.44-77.57)	0.66	0.82 (0.57-1.05)	1.25 (0.89-1.58)	0.99 (0.74-1.24)
Luxembourg	6.35 (4.99-7.55)	6.34 (4.91-7.75)	0	1.22 (0.97-1.45)	0.61 (0.47-0.74)	-2.1 (-2.22--1.99)
Macedonia	12.13 (8.99-15.68)	17.43 (12.11-22.85)	0.44	0.62 (0.46-0.82)	0.52 (0.37-0.69)	-0.74 (-0.89--0.59)
Madagascar	17.41 (6.91-27)	21.84 (8.27-37.78)	0.25	0.31 (0.13-0.49)	0.16 (0.06-0.27)	-2.43 (-3.01--1.85)
Malawi	6.79 (3.14-10.55)	22.72 (13.42-33.47)	2.35	0.16 (0.08-0.2	0.26 (0.15-0.3	1.69 (1.62-1.76)

				5)	8)	
Malaysia	25.27 (15.03-37.58)	38.53 (20.83-60.15)	0.52	0.26 (0.15-0.38)	0.13 (0.07-0.2)	-2.93 (-3.42--2.44)
Maldives	0.02 (0-0.08)	0.16 (0.05-0.35)	7	0.02 (0-0.09)	0.04 (0.01-0.09)	-2.16 (-4.3-0.03)
Mali	2.99 (1.58-4.55)	7.63 (4.18-11.75)	1.55	0.07 (0.04-0.11)	0.08 (0.05-0.13)	0.45 (0.4-0.51)
Malta	1.82 (1.3-2.37)	2.97 (2.18-3.87)	0.63	0.43 (0.31-0.56)	0.35 (0.26-0.45)	-0.63 (-0.77--0.48)
Marshall Islands	0.02 (0.01-0.04)	0.07 (0.03-0.13)	2.5	0.1 (0.04-0.18)	0.18 (0.08-0.31)	2 (1.9-2.09)
Mauritius	3.98 (2.64-5.11)	8.7 (5.93-11.38)	1.19	0.52 (0.34-0.67)	0.47 (0.32-0.61)	-0.32 (-0.73-0.09)
Mexico	91.48 (69.34-113.43)	226.86 (165.97-293.85)	1.48	0.21 (0.16-0.27)	0.18 (0.13-0.23)	-0.81 (-1.02--0.6)
Micronesia (Federated States of)	0.13 (0.06-0.21)	0.15 (0.07-0.24)	0.15	0.24 (0.12-0.4)	0.17 (0.08-0.28)	-1.44 (-1.55--1.33)
Moldova	58.8 (43.49-72.07)	60.58 (44.06-76.81)	0.03	1.28 (0.94-1.57)	1.04 (0.76-1.31)	-1.09 (-1.45--0.73)
Monaco	0.11 (0-0.22)	0.17 (0-0.34)	0.55	0.19 (0-0.36)	0.2 (0.01-0.39)	0.25 (0.17-0.33)
Mongolia	3.69 (1.94-5.86)	11.01 (6.84-15.97)	1.98	0.33 (0.17-0.52)	0.41 (0.25-0.61)	1.53 (1.24-1.82)
Montenegro	4.83 (3.4-6.37)	7.5 (5.37-9.69)	0.55	0.74 (0.52-0.97)	0.79 (0.57-1.03)	0.17 (0.03-0.3)
Morocco	1.24 (0.76-1.86)	2.18 (1.2-3.56)	0.76	0.01 (0-0.01)	0.01 (0-0.01)	-0.66 (-0.76--0.55)
Mozambique	1.97 (0.15-4.66)	20.97 (9.26-33.73)	9.64	0.03 (0-0.07)	0.17 (0.07-0.27)	5.69 (5.51-5.86)
Myanmar	7.23 (2.08-16.42)	115.77 (70.34-172.2)	15.01	0.03 (0.01-0.0	0.23 (0.14-0.3	8.35 (7.83-8.87)

				6)	4)	
Namibia	6.17 (2.91-9.74)	25.11 (15.69-35.88)	3.07	0.87 (0.41-1.4)	1.64 (1.04-2.32)	2.18 (1.91-2.45)
Nauru	0.01 (0.01-0.03)	0.03 (0.01-0.05)	2	0.28 (0.11-0.51)	0.37 (0.19-0.68)	1.22 (1.09-1.36)
Nepal	3.23 (0.09-12.32)	145.45 (55.87-257.18)	44.03	0.03 (0-0.11)	0.59 (0.22-1.04)	9.77 (8.39-11.17)
Netherlands	115.2 (88.14-140.01)	153.76 (117.75-187.39)	0.33	0.6 (0.46-0.72)	0.44 (0.34-0.54)	-0.88 (-1.06--0.7)
New Zealand	25.44 (16.47-33.13)	38.49 (30.03-47.4)	0.51	0.66 (0.43-0.86)	0.46 (0.36-0.57)	-0.84 (-1.16--0.52)
Nicaragua	1.46 (1-1.97)	4.79 (3.32-6.51)	2.28	0.09 (0.06-0.12)	0.09 (0.06-0.13)	0.45 (0.19-0.71)
Niger	0.07 (0-0.23)	0.76 (0.15-1.87)	9.86	0 (0-0.01)	0.01 (0-0.02)	5.76 (4.7-6.83)
Nigeria	46.33 (27.74-65.27)	131.87 (86.53-187.99)	1.85	0.1 (0.06-0.14)	0.14 (0.09-0.19)	0.99 (0.9-1.08)
Niue	0 (0-0.01)	0.01 (0-0.01)	Inf	0.2 (0.03-0.33)	0.3 (0.15-0.46)	0.84 (0.53-1.15)
Northern Mariana Islands	0.07 (0-0.16)	0.5 (0.05-0.89)	6.14	0.27 (0-0.65)	0.87 (0.09-1.59)	4.28 (3.91-4.66)
Norway	23.45 (15.63-31.47)	29.62 (21.9-37.75)	0.26	0.38 (0.26-0.5)	0.3 (0.23-0.38)	-0.72 (-0.97--0.46)
Oman	0.12 (0.05-0.22)	0.42 (0.21-0.72)	2.5	0.01 (0.01-0.03)	0.01 (0.01-0.02)	-0.33 (-1.47-0.82)
Pakistan	101.79 (19.81-193.05)	549.53 (233.43-913.78)	4.4	0.16 (0.03-0.3)	0.36 (0.14-0.61)	2.86 (2.57-3.15)
Palau	0.14 (0.02-0.31)	0.44 (0.11-0.85)	2.14	1.29 (0.15-2.79)	1.79 (0.47-3.42)	1.06 (0.95-1.17)
Palestine	0.19 (0.09-0.3)	0.49 (0.3-0.75)	1.58	0.02 (0.01-0.0	0.02 (0.01-0.0	-0.88 (-1.1--0.66)

				3)	3)	
Panama	4.66 (3.44-5.96)	10.37 (6.78-14)	1.23	0.31 (0.23-0.4)	0.23 (0.15-0.32)	-0.8 (-0.99--0.61)
Papua New Guinea	1.62 (0.72-2.7)	4.79 (2.23-7.78)	1.96	0.07 (0.03-0.12)	0.08 (0.03-0.12)	0.7 (0.06-1.33)
Paraguay	10.96 (7.69-14.48)	33.26 (21.74-48.32)	2.03	0.48 (0.34-0.64)	0.55 (0.36-0.8)	0.23 (0.1-0.36)
Peru	23.48 (14.26-33.98)	57.22 (36.18-86.47)	1.44	0.19 (0.11-0.27)	0.17 (0.11-0.26)	-0.17 (-0.59-0.24)
Philippines	146.32 (101.7-193.39)	378.01 (269.58-503.23)	1.58	0.46 (0.32-0.61)	0.43 (0.31-0.58)	-0.25 (-0.33--0.18)
Poland	369.27 (282.21-452.38)	753.68 (596.49-919.63)	1.04	0.86 (0.66-1.06)	1.15 (0.91-1.38)	0.98 (0.88-1.08)
Portugal	149.77 (116.77-177.74)	179.55 (139.14-223.25)	0.2	1.13 (0.89-1.34)	0.85 (0.68-1.04)	-0.93 (-1.07--0.79)
Puerto Rico	23.1 (16.43-29.61)	17.36 (11.81-23.6)	-0.25	0.64 (0.46-0.82)	0.28 (0.19-0.37)	-2.35 (-2.58--2.11)
Qatar	0.03 (0.02-0.05)	0.27 (0.13-0.44)	8	0.02 (0.01-0.03)	0.02 (0.01-0.03)	0.03 (-0.7-0.76)
Romania	247.3 (189.1-305.89)	476.72 (369.4-598.36)	0.93	0.88 (0.67-1.1)	1.45 (1.12-1.83)	1.7 (1.4-2)
Russian Federation	1685.58 (1251.59-2047.26)	1928.57 (1400.56-2428.47)	0.14	0.92 (0.69-1.11)	0.85 (0.64-1.06)	-0.96 (-1.3--0.63)
Rwanda	33.15 (19.16-47.47)	48.66 (26.7-75.75)	0.47	1.07 (0.62-1.51)	0.69 (0.38-1.06)	-2.18 (-2.45--1.92)
Saint Kitts and Nevis	0.16 (0-0.22)	0.18 (0-0.43)	0.12	0.44 (0-0.62)	0.24 (0-0.59)	-1.72 (-2.1--1.33)
Saint Lucia	0.91 (0.7-1.12)	1.82 (1.29-2.4)	1	1.05 (0.8-1.31)	0.75 (0.53-0.99)	-1.08 (-1.3--0.86)
Saint Vincent and the Grenadines	0.57 (0.38-0.77)	1.63 (1.19-2.11)	1.86	0.8 (0.54-1.0	1.13 (0.83-1.4	1.35 (1.01-1.69)

				8)	6)	
Samoa	0.19 (0.09-0.28)	0.23 (0.11-0.38)	0.21	0.2 (0.09-0.3)	0.15 (0.07-0.24)	-1.29 (-1.66--0.92)
San Marino	0.19 (0-0.3)	0.21 (0-0.37)	0.11	0.57 (0-0.88)	0.3 (0-0.53)	-1.09 (-1.43--0.75)
Sao Tome and Principe	0.01 (0-0.01)	0.02 (0.01-0.02)	1	0.01 (0.01-0.01)	0.01 (0.01-0.02)	0.74 (0.41-1.07)
Saudi Arabia	0.96 (0.25-1.77)	1.78 (0.23-3.76)	0.85	0.01 (0-0.03)	0.01 (0-0.01)	-1.24 (-1.87--0.6)
Senegal	0.76 (0.18-1.56)	1.52 (0.37-3.37)	1	0.02 (0.01-0.04)	0.02 (0-0.04)	-1.25 (-1.65--0.85)
Serbia	99.21 (65.47-135.69)	118.24 (83.93-154.48)	0.19	0.85 (0.55-1.18)	0.78 (0.56-1.01)	-0.3 (-0.42--0.18)
Seychelles	0.62 (0.42-0.83)	1.63 (1.14-2.14)	1.63	1.11 (0.76-1.48)	1.32 (0.92-1.74)	0.51 (0-1.02)
Sierra Leone	2.79 (1.6-3.99)	6.07 (3.67-9.24)	1.18	0.13 (0.08-0.19)	0.15 (0.09-0.22)	0.12 (0.02-0.23)
Singapore	2.59 (1.72-3.53)	5.58 (3.77-7.53)	1.15	0.11 (0.07-0.14)	0.06 (0.04-0.09)	-2.17 (-2.71--1.63)
Slovakia	138.98 (107.14-174.21)	140.46 (95.93-192.22)	0.01	2.41 (1.86-3)	1.57 (1.07-2.13)	-1.46 (-1.52--1.4)
Slovenia	26.23 (19.23-32.7)	16.55 (8.38-25.47)	-0.37	1.06 (0.78-1.33)	0.41 (0.21-0.63)	-3.47 (-3.68--3.26)
Solomon Islands	0.04 (0.01-0.07)	0.29 (0.12-0.55)	6.25	0.02 (0-0.04)	0.07 (0.02-0.12)	5.29 (4.36-6.23)
South Africa	208.66 (126.46-282.43)	366.46 (255.09-459.89)	0.76	0.95 (0.57-1.3)	0.74 (0.52-0.93)	-1.28 (-1.51--1.05)
South Korea	104.09 (79.92-132.08)	224.52 (158.2-293.36)	1.16	0.33 (0.25-0.42)	0.25 (0.17-0.32)	-1.46 (-1.75--1.18)
South Sudan	0.72 (0.02-1.87)	0.7 (0.08-1.96)	-0.03	0.03 (0-0.07)	0.01 (0-0.04)	-1.68 (-1.96--1.41)
Spain	544.05	507.23	-0.07	1.07	0.56	-2.44

	(417.54-659.68)	(397.7-624.44)		(0.84-1.3)	(0.44-0.69)	(-2.59--2.3)
Sri Lanka	54.28 (35-74.14)	201.08 (107.88-316.33)	2.7	0.51 (0.32-0.69)	0.73 (0.39-1.14)	1.78 (1.39-2.17)
Sudan	4.74 (2.78-7.93)	0 (0-0.01)	-1	0.05 (0.03-0.08)	0 (0-0)	-21.56 (-26.31--16.49)
Suriname	0.8 (0.56-1.04)	1.47 (0.93-2.27)	0.84	0.3 (0.21-0.39)	0.22 (0.14-0.34)	-0.5 (-0.69--0.31)
Swaziland	2.01 (1.29-2.94)	4.05 (2.21-6.67)	1.01	0.63 (0.42-0.92)	0.64 (0.35-1.04)	-0.04 (-0.36-0.28)
Sweden	60.01 (44.71-74.41)	77.66 (56.52-98.3)	0.29	0.43 (0.33-0.53)	0.37 (0.28-0.47)	0.2 (-0.11-0.51)
Switzerland	63.41 (48.92-77.38)	75.28 (56.81-94.16)	0.19	0.65 (0.52-0.79)	0.43 (0.33-0.53)	-1.39 (-1.68--1.09)
Syria	1.21 (0.7-1.88)	1.37 (0.61-2.34)	0.13	0.02 (0.01-0.03)	0.01 (0-0.02)	-2.71 (-3.11--2.29)
Taiwan (Province of China)	140.55 (111.75-168.01)	599.08 (467.55-760.7)	3.26	0.81 (0.64-0.97)	1.51 (1.18-1.91)	1.57 (1.04-2.1)
Tajikistan	2.33 (1.35-3.61)	3.27 (1.9-5.21)	0.4	0.08 (0.04-0.12)	0.04 (0.02-0.07)	-2.17 (-2.42--1.91)
Tanzania	77.78 (43.62-114.14)	162.71 (99.13-240.52)	1.09	0.68 (0.38-0.98)	0.6 (0.36-0.88)	-0.56 (-0.62--0.49)
Thailand	259.13 (180.36-348.45)	634.71 (443.69-898.11)	1.45	0.68 (0.47-0.91)	0.6 (0.42-0.84)	-0.77 (-0.89--0.65)
The Bahamas	1.52 (1.13-1.92)	2.45 (1.37-3.59)	0.61	0.92 (0.68-1.18)	0.56 (0.31-0.83)	-1.49 (-1.73--1.24)
The Gambia	0.23 (0.08-0.42)	1.58 (0.87-2.48)	5.87	0.06 (0.02-0.11)	0.15 (0.08-0.23)	2.77 (2.21-3.33)
Timor-Leste	0.37 (0.16-0.64)	2.06 (1.22-3.05)	4.57	0.12 (0.05-0.2)	0.24 (0.14-0.35)	2.04 (1.78-2.31)

Togo	1.44 (0.74-2.19)	5.94 (3.2-9.41)	3.12	0.1 (0.06-0.16)	0.13 (0.07-0.21)	0.92 (0.66-1.17)
Tokelau	0 (0-0)	0 (0-0.01)	NA	0.13 (0.05-0.24)	0.23 (0.1-0.38)	1.96 (1.9-2.03)
Tonga	0.04 (0.01-0.06)	0.05 (0.01-0.1)	0.25	0.06 (0.02-0.11)	0.06 (0.02-0.12)	-0.27 (-1.1-0.57)
Trinidad and Tobago	3.34 (2.38-4.31)	6.09 (4.16-8.85)	0.82	0.39 (0.28-0.51)	0.31 (0.21-0.46)	-0.34 (-0.53--0.16)
Tunisia	3.63 (2.2-5.3)	10.5 (6.17-16.42)	1.89	0.07 (0.04-0.1)	0.08 (0.04-0.12)	0.19 (0.08-0.29)
Turkey	24.16 (14.55-35.54)	45.31 (26.76-67.8)	0.88	0.06 (0.04-0.09)	0.05 (0.03-0.07)	-1.59 (-1.86--1.32)
Turkmenistan	6.87 (3.59-10.77)	20.94 (13.05-29.43)	2.05	0.32 (0.16-0.51)	0.47 (0.29-0.66)	0.95 (0.68-1.22)
Tuvalu	0.01 (0-0.01)	0.02 (0.01-0.03)	1	0.08 (0.03-0.15)	0.14 (0.06-0.25)	1.7 (1.26-2.16)
Uganda	48.42 (24.96-68.4)	152.84 (94.69-216.82)	2.16	0.68 (0.37-0.96)	0.89 (0.56-1.26)	0.46 (0.25-0.67)
Ukraine	529.55 (362.52-712.58)	667.07 (390.25-994.88)	0.26	0.74 (0.51-0.99)	0.94 (0.55-1.39)	0.91 (0.44-1.38)
United Arab Emirates	1.36 (0.77-2.27)	6.18 (3.7-9.53)	3.54	0.19 (0.1-0.34)	0.1 (0.05-0.16)	-1.31 (-1.64--0.98)
United Kingdom	433.09 (323-534.92)	703.76 (555.61-843.78)	0.62	0.52 (0.39-0.63)	0.58 (0.46-0.69)	0.4 (0.31-0.49)
United States	1478 (974.46-1987.14)	2238.01 (1680.2-2781.15)	0.51	0.49 (0.33-0.65)	0.39 (0.3-0.48)	-0.53 (-0.75--0.32)
Uruguay	32.81 (25.53-40.93)	35.11 (27.03-44.74)	0.07	0.86 (0.67-1.08)	0.68 (0.53-0.86)	-0.7 (-0.81--0.59)
Uzbekistan	20.2 (11.93-28.99)	74.6 (47.13-103.87)	2.69	0.16 (0.09-0.2	0.25 (0.16-0.3	1.12 (0.86-1.37)

				3)	5)	
Vanuatu	0.07 (0.03-0.13)	0.3 (0.16-0.5)	3.29	0.1 (0.05-0.16)	0.15 (0.07-0.24)	1.89 (0.66-3.14)
Venezuela	34.82 (27.13-42.98)	74.88 (48.48-110.28)	1.15	0.35 (0.27-0.43)	0.25 (0.16-0.36)	-1.31 (-1.53--1.1)
Viet Nam	12.71 (1.75-31.55)	969.23 (671.27-1305.85)	75.26	0.03 (0-0.07)	0.92 (0.64-1.23)	11.26 (9.72-12.83)
Virgin Islands, U.S.	0.32 (0-0.65)	0.46 (0.02-0.86)	0.44	0.36 (0-0.74)	0.28 (0.01-0.51)	-0.69 (-0.91--0.46)
Yemen	1.68 (0.85-2.87)	1.34 (0.74-2.31)	-0.2	0.03 (0.01-0.05)	0.01 (0-0.01)	-4.97 (-5.27--4.67)
Zambia	16.94 (8.22-24.55)	87.91 (32.01-153.95)	4.19	0.54 (0.26-0.78)	1.04 (0.41-1.81)	2.78 (2.17-3.4)
Zimbabwe	14.76 (8.18-20.69)	34.16 (20.27-50.02)	1.31	0.34 (0.18-0.47)	0.42 (0.25-0.61)	0.83 (0.59-1.06)

Supplementary table 2. The deaths of lip and oral cavity cancer attributable to high alcohol consumption cases and rates in 1990 and 2021 across 204 countries, and the trends from 1990 to 2021.
